# Epidemiology and Molecular Biology of HPV Variants in Cervical Cancer: The State of the Art in Mexico

**DOI:** 10.3390/ijms23158566

**Published:** 2022-08-02

**Authors:** J. Omar Muñoz-Bello, Adela Carrillo-García, Marcela Lizano

**Affiliations:** 1 Unidad de Investigación Biomédica en Cáncer, Instituto Nacional de Cancerología, Av. San Fernando No. 22, Col. Sección XVI, Tlalpan, Mexico City 14080, Mexico; omarmube@gmail.com (J.O.M.-B.); adcarrillo2004@yahoo.com.mx (A.C.-G.); 2 Departamento de Medicina Genómica y Toxicología Ambiental, Instituto de Investigaciones Biomédicas, Universidad Nacional Autónoma de México, Circuito Exterior S/N, Ciudad Universitaria, Mexico City 04510, Mexico

**Keywords:** HPV, variants, cervical cancer, Mexico

## Abstract

Cervical cancer (CC) continues to be a major public health problem in Mexico, ranking second among cancers in women. A persistent infection with human papillomaviruses (HPV) is the main risk factor for CC development. In addition, a significant fraction of other cancers including those of the anus, oropharynx, and penis are also related to HPV infection. In CC, HPV-16 is the most prevalent high-risk HPV type, followed by HPV-18, both being responsible for 70% of cases. HPV intratype variant lineages differ in nucleotide sequences by 1–10%, while sublineages differ by 0.5–1%. Several studies have postulated that the nucleotide changes that occur between HPV intratype variants are reflected in functional differences and in pathogenicity. Moreover, it has been demonstrated that HPV-16 and -18 intratype variants differentially affect molecular processes in infected cells, changing their biological behavior that finally impacts in the clinical outcome of patients. Mexico has participated in providing knowledge on the geographical distribution of intratype variants of the most prevalent HPVs in premalignant lesions of the cervix and cervical cancer, as well as in other HPV-related tumors. In addition, functional studies have been carried out to assess the cellular effects of intratype variations in HPV proteins. This review addresses the state of the art on the epidemiology of HPV-16 and HPV-18 intratype variants in the Mexican population, as well as their association with persistence, precancer and cervical cancer, and functional aspects related to their biological behavior.

## 1. Introduction

Among the seven known human viruses associated with cancer, high-risk human papillomaviruses (HR-HPV) cause the greatest cancer burden, with more than 690,000 cancer cases attributed to HPV in 2018 [1]. Cervical cancer (CC) is the most common neoplasia related to HPV infections. A persistent infection with HR-HPV is the main risk factor for the development of most CCs (>85%) [2,3]. Additionally, other anogenital cancers, such as anal, vulvar, vaginal, penile, and a proportion of oropharyngeal and oral cancers, are also caused by HPV [4,5]. Therefore, HPV-related cancers affect both the female and the male populations.

Cervical cancer continues to be a public health problem, and it ranks fourth among cancers in women worldwide [6]. In Mexico, CC ranks second among cancers in women, where 9439 new cases and 4335 deaths from this neoplasia were estimated in 2020 [7].

Human papillomaviruses (HPV) are small non-enveloped particles which infect skin and mucous membranes. Virus particles contain approximately 8000 bp of double-stranded DNA, covered by a viral capsid composed of L1 and L2 proteins. According to their genomic sequence similarity, more than 200 types of HPV have been described [8], considering that each type of HPV differs from another by at least 10% in the highly conserved L1 gene [9]. Based on such similarity, HPVs are divided into five genera (α, β, γ, μ, and ν), where α-papillomaviruses predominantly contain HPV types that infect epithelial mucosa [10]. In addition, HPVs are classified into high-risk (HR) or low-risk (LR) types based on their oncogenic potential. Approximately 20 high-risk types can be found in HPV-related cancers, although HPV-16, is the most common type found in cervical cancer (50%) and premalignant lesions of the cervix as well as in 80% of oropharyngeal cancers [11,12,13,14]. The high prevalence of HPV-16 in cancer in relation to other oncogenic HPVs may be due to a greater oncogenicity, partly explained by the differential effects exerted by HPV-16 oncogenes on cellular targets. After HPV-16, the second most carcinogenic HPV type is HPV-18, which is found in approximately 12% of squamous cell carcinomas and 37% of cervical adenocarcinomas worldwide [15].

Sequence diversity is also found within each HPV type, where intratype variant lineages differ by 1–10%, while sublineages differ by 0.5–1% [16,17]. Being double-stranded DNA viruses, papillomaviruses use the efficient proofreading DNA polymerase of the host for their replication, which avoids high mutation rates; therefore, changes in HPV genomes have been acquired slowly, defining HPV types whose infection cycle has adapted to different host cells. Random mutations that eventually occur within viral types have generated the nucleotide polymorphisms contained in HPV intratype variants. When the intratype variants of HPV-16 and -18, began to be studied, they were named African, European, Asian, Asian-Amerindian, depending on the geographical origin and ethnicity of the infected population. Subsequently, several studies showed that HPV variants are spread throughout the world, and a more precise denomination was proposed, changing the nomenclature to lineages and sublineages [17].

Many HPV intratype variants have been isolated in various clinical studies and a careful classification of variants of the most frequent HPV types in cancer has been achieved [9,15,17]. It has been postulated that nucleotide variations that occur among HPV intratype variants are reflected in functional differences and in their pathogenicity [18,19,20,21]. Currently, there are many studies that report that HPV-16 and -18 intratype variants differentially alter host cells, which translates into different risks of persistence and progression to cancer [22,23,24,25,26,27,28,29].

Several epidemiological studies have been carried out on the geographical distribution of HPV types and intratype variants worldwide and Mexico is not the exception. In addition, functional studies have also been carried out regarding the participation of viral proteins and the transcriptional activity of the viral non-coding region with the goal of identifying differences in biological behavior towards the establishment of cancer, mainly focused on HPV-16 and -18 intratype variants.

Deciphering the impact that HPV intratype variations have on cancer development, as well as on the clinical outcome of patients with HPV-related cancers, could eventually influence patient prognosis and the development of therapeutic strategies. This review addresses the state of the art on the epidemiology of HPV-16 and HPV-18 intratype variants worldwide and particularly in the Mexican population, as well as their association with persistence, precancer and cervical cancer, and functional aspects related to their biological behavior.

## 2. HPV Infection and Life Cycle

The human papillomavirus genome consists of three functionally different regions: an early expressed region (E) that includes genes E1, E2, E4, E5, E6, and E7; a late expressed region (L) that contains the L1 and the L2 genes; and a long non-coding region (LCR) that contains elements that regulate viral replication and transcription [30].

The HPV replicative cycle is dependent on the differentiation process of the epithelium. During the initial HPV infection, E viral genes are expressed from the undifferentiated basal epithelium where E1 and E2 products regulate viral genome replication and E2 controls the expression of E6 and E7 oncoproteins. In addition, E6 and E7 oncoproteins interact with a variety of cellular proteins that favor the replication of the viral genome. L1 and L2 proteins are produced in the most differentiated layers of the epithelium and after being assembled, virions are released by cellular desquamation in the most superficial layers.

To establish a productive infection, HPV must infect the basal cells of a differentiating epithelium, where the viral genome is amplified and subsequently packaged, allowing for the generation of new viral particles. Through micro-wounds, HPVs reach and infect mitotically active basal cells that will eventually divide and enter the parabasal layer, undergoing differentiation. A specific receptor that mediates the entry of the virus into the host cell has not yet been described, although different receptors have been proposed [31]. HPV entry into the cell occurs by endocytosis independent of clathrin, caveolin, lipid raft, and dynamin [32]. It has been proposed that HPV binds to cell-free Heparan Sulfate (HS) or Heparan Sulfate Syndecan (Sdc) proteoglycan (HPSG), Sdc2, and Sdc4, bound to the cell membrane [33]. In addition, cell membrane receptors have been identified, including EGFR [34], α6-integrin [35], CD63 [36] and CD151 tetraspannin [37], and annexin A2/S100A10 heterotetramer (A2t), which are required for HPV uptake [38].

HPV interaction with HPSG exposes L2 on the capsid surface and mediates conformational changes in L1, allowing the kallikrein-8 (KLK8) protease to cleave L1, thereby exposing the RG-1 epitope within the N-terminus of the L2 protein [39]. Furin protease then cleaves L2 upstream of the RG-1 epitope [40], preparing the viral particle for entry and proper downstream trafficking of L2 that binds to the HPV episome. L2 then binds to γ-secretase protease and p120-catenin that act as chaperones of L2, allowing insertion of L2 into vesicular membranes [41,42]. Once in the endosome, L2 interacts with different proteins that ensure vesicular trafficking of the L2-HPV episome. For example, L2 has been shown to interact with Sortin Nexin 17 (SNX17), which prevents vesicle acidification and rapid lysosomal degradation of vesicle content, hence the L2-episome maintains its integrity [43]. Additionally, L2 binds to members of the retromer cargo complex, including Vps26, Vps29, and Vps35 [44], essential for retrograde trafficking of L2-HPV episomes to the Trans Golgi Network (TGN). Then, L2-HPV episomes exit the endosomal compartments and traffic to the TGN where they remain until the onset of mitosis [31]. During mitosis, the spindle microtubule is required to transport the L2-HPV episome, reaching the mitotic chromosomes and centrosomes, since L2 interacts with motor proteins Ran-binding protein 10 (RanBP10), Karyopherin alpha2 (KPNA2), and dynein light chain (DYNLT3) [45,46]. Subsequently, L2 binds to the cellular mitotic chromosomes, allowing L2-dependent chromosomal anchoring of the L2-HPV episome during open mitosis [47]. Finally, the viral episome is delivered into the nucleus of daughter cells in a mitosis-dependent manner, where HPV episomes are localized to highly transcriptionally active sites termed ND10/PML bodies [48].

Once inside the nucleus, HPV transcription is driven by the p97 and p105 promoters of HPV-16 and -18, respectively, allowing the expression of E6, E7, E1, and E2 genes, necessary for the early stages of viral genome amplification. E2 protein contains a DNA binding domain, and once it binds to its response elements located in the LCR, it negatively regulates the expression of the early expressed viral genes [49]. Meanwhile, E1 protein acts as an ATP-dependent DNA helicase, and it is considered the only enzyme encoded by papillomaviruses [50]. E2 interacts with E1, forming hexameric dimers that bind to the origin of replication and unwind the DNA ahead the replication fork, allowing recruitment of the replication machinery. The viral episomes are amplified in a low number of copies (50–100 episomes per nucleus) [51], which favors the evasion of immunological surveillance and, consequently, the establishment of persistent infections. Additionally, it has been shown that E1 proteins suppress the expression of genes related to immune response, such as IFNβ1 and IFNλ1 and Interferon-Stimulated Genes (ISG) [52].

Importantly, the expression of E6 and E7 is necessary for infected cells to enter mitosis, and the amplification of the HPV genome partially depends on the cell division program. Although the activity of E6 and E7 oncoproteins is crucial for the establishment of cancer, their expression is also necessary to carry out the replicative cycle of HPV.

E7 is responsible for maintaining the continuity of the cell cycle, promoting the transition of the G1 to S phase, since E7 interacts with the tumor suppressor pRb and with the E3 ubiquitin ligase Cullin 2, which induces the degradation of pRb [53]. These events allow the release of the transcriptional factor E2F from its inhibitor, pRb, promoting the expression of target genes, such as cyclin E, cyclin A, and p16INK4A, among others [53].

Generally, E6 and E7 proteins are produced from a bicistronic mRNA and the activities of both viral oncogenes in HPV-infected cells ensure the generation of viral progeny. In this context, it may be that when E7 induces excessive proliferation, then E6 could contribute by mitigating such effect.

It has been described that E6 interacts with the tumor suppressor p53, forming a complex with E6AP (E6-associated protein), promoting the degradation of p53 through the proteasome. This event impedes p53 functions, such as induction of apoptosis and DNA damage repair [53].

In addition, E6 and E7 regulate different signaling pathways, such as Wnt/β-catenin [54], PI3K/Akt [55], and Notch [56], among others, which together contribute to the promotion of the viral replicative cycle.

Furthermore, viral E5 protein maintains cell proliferation in the upper layers of the epithelium. E5 prevents endosomal acidification by binding to the 16-kDa subunit of vacuolar ATPase (vATPase), which allows the continuous membrane recycling of the EGFR receptor, producing its activation and the activation of downstream effectors. In addition, E5 promotes immune system evasion by preventing antigen presentation by the MHCI, since it indirectly interacts with the heavy chain of MHCI, preventing its correct trafficking to the membrane resulting in its accumulation in the Golgi apparatus and endoplasmic reticulum [57]. At this point of the infection, HPV episomes increase up to thousands of copies per cell.

Meanwhile, the E4 protein has been shown to promote the collapse of the cytokeratin network, which makes cells fragile and more likely to release progeny virions [58]. Moreover, keratin has been shown to be hyperphosphorylated at residues K8 and S73, as well as ubiquitinated in the presence of E4, suggesting that its degradation could be promoted through the proteasome, allowing network disruption [59].

Finally, viral capsid proteins are synthesized from the late promoter p670 and p811 for HPV-16 and -18, respectively, and assembled in terminally differentiated cells. The HPV genome is encapsidated in an icosahedral outer shell made up of 360 L1 molecules organized into 72 pentameric capsomeres, where L2 is localized at the center [60].

During the infection process, reactive oxygen species, genomic instability, and DNA strand breaks are generated, which can favor the integration of the viral genome into the cellular genome. This integration process promotes the deregulated expression of E6 and E7 oncogenes, which is considered an initial step for cervical carcinogenesis. The interaction of E6 and E7 with tumor suppressors and with proteins involved in cellular signaling pathways alter normal cell function, leading to transformation [53,61].

## 3. HPV Pathogenesis

Most women will be infected by HPV at least once in their lives [62]; however, it has been reported that about 90% of these HPV infections are transient and will disappear within the first two years due to the host immune system [63]. Although progression to cancer is relatively rare, a persistent HPV infection is a key event determining progression to cervical cancer. Therefore, cancer caused by HPV is a sporadic event which is not beneficial for the viral replicative cycle or for the generation of viral progeny.

A minority of infections become persistent (10–20%) and are at increased risk of progression to cancer [64]. It has been demonstrated that in women who were persistently infected with one HR-HPV and tested over 2 years, the risk of cervical cancer was 12.4% compared to 0.14% in women with a repeatedly negative HPV test. Moreover, the risk of developing cancer increases with age, being 5.5%, 14.4%, and 18.1% for women aged 30 to 44 years, 45 to 54 years, and 55 years or older, respectively [65].

Among the factors proposed to determine persistence that are inherent to the virus are viral load, viral type, and intratype variations, while those inherent to the host include genetic predisposition and lifestyle factors [66].

Prior to the establishment of cancer, a series of clinical characteristics inherent to HPV infection occur, where the normal epithelium evolves into epithelial precursor lesions, causing cervical disease. Such lesions have been histologically classified into three groups, according to the progression of the lesion: cervical intraepithelial neoplasia (CIN) 1, 2, and 3 (mild, moderate, and severe epithelial dysplasia, respectively). Additionally, another classification based in cytological evaluation, divides the lesions into two groups known as low-grade squamous intraepithelial lesions (mild dysplasia) (LGSIL) and high-grade squamous intraepithelial lesions (moderate to severe dysplasia) (HGSIL) [67].

CIN1 or LGSIL lesions have a low probability of progressing to cervical cancer and are caused by transient HPV infections and 90% of them revert to healthy epithelium. Conversely, CIN2 and 3 or HGSIL are caused by persistent and unproductive HPV infections, yet 60% of such lesions clear spontaneously in immunologically competent individuals [68]. In this scenario, 0.6% of HPV infections are known to progress to cancer. A study with 16 years follow-up showed that women with persistent infections with any type of carcinogenic HPV have a 75.4-fold increased risk of developing cancer compared to HPV-negative women [65]. Interestingly, some HPV-16 and -18 variants have been associated with a high risk of persistence, which may be influencing the clinical outcome of the infection.

## 4. Classification of HPV-16 and -18 Lineages and Sublineages

Among the first studies that described the existence of intratype variants of HPV-16, that performed in 1993 by Ho et al. [69] stands out. Based on the LCR sequence, they classified intratype variants into five main branches, named according to the geographical origin of the populations where the variants were found with a higher prevalence: European (E), Asian (As), Asian-American (AA), and two African lineages (Af1 and Af2). In 1997, Yamada et al. [70,71] in addition to the LCR, included sequences from E6 and L1 genes for their classification, thereby identifying an additional North American lineage (NA). Later, Cornet I et al. (2012) [72] used 13 nucleotide positions in the E6 gene and 32 positions in the LCR, and identified nine distinct sublineages of HPV-16 (EUR, As, Af1a, Af1b, Af2a, Af2b, NA, AA1, and AA2). Most recent studies, including the complete viral genome, identified 4 main lineages (A, B, C, D) of HPV-16 with 16 sublineages, including A1-3 (previously European), A4 (Asian), B1-4 (African1), C1-4 (Af-2), D1 (North American), D2-3 (Asian-American) and D4 [17,22,73,74,75].

HPV-18 variants were initially classified according to nucleotide changes in the L1 gene as European (E), Asian-Amerindian (AsAi), and African (Af) [76]. Subsequently, through phylogenetic analysis of the complete LCR and the E6 gene, the HPV-18 variants were grouped into 3 lineages (A, B and C) and 9 sublineages, where A1/A2 were previously AsAi variants; A3/A4, were the European variants; and the B sublineages were formerly the African variants [15].

According to the Papillomavirus Episteme (PaVE) portal [74], the E6 variants of HPV-16 contain at least 17 nucleotide changes in their sequence with respect to the reference variant (A1), while in E7 at least 7 mutations have been observed. Meanwhile, for HPV-18, at least 14 and 9 mutations have been reported in the E6 and E7 variants, respectively, relative to the reference variant.

Regarding HPV-16, the greatest number of variations are detected in the B4 sublineage with 7 nucleotide substitutions in the E6 gene, impacting in 4 amino acid changes. Meanwhile for the E7 gene, sublineages B2, C1, C4, D2 and D3 present 3 mutations each. In the E7 protein, the A4, B2, C1 and C4 sublineages were affected with one amino acid change. In relation to HPV-18, the B1 sublineage is the most different in relation to the A1 reference sublineage, with 9 nucleotide changes in E6 gene and 2 amino acid substitutions; while in the E7 gene, B1, B2 and B3 sublineages exhibit 4 sequence mutations. Interestingly, the B2 sublineage contains 3 amino acid substitutions in the E7 protein, being the most different variant. The nucleotide and amino acid alignments of HPV-16 and -18 sublineages available in PaVE are described in detail in the Supplementary Material.

## 5. HPV-16 and -18 Intratype Variants and Risk of Persistence or Cervical Cancer

The distribution of HPV intratype variants varies significantly in different geographical areas [23,77,78,79]. Nevertheless, there is accumulating evidence showing that non-European HPV-16 variants, have a higher oncogenicity due to their association with high-grade lesions of the cervix and invasive tumors [22,23,24,27,80,81,82,83].

In regard to HPV-18, several studies, although controversial, support an association of certain sublineages with infection persistence or CC risk. A Brazilian study shows that HPV-18 European variants are more likely to develop persistent infections [26]; in contrast, a study with a Portuguese population indicates that HPV-18 European variants are associated with a lower risk for CC development [84]. Xi et al. (2007) [85] stated that the risk of developing > CIN3 was higher for European and Asian-American variants, compared with HPV-18 Af variants. Another study, which analyzed cervical samples from the International Agency for Research on Cancer (IARC) biobank collected from different regions worldwide, affirms that HPV-18 variant sublineages cannot discriminate cancer risk [15].

Furthermore, it has been determined that some HPV-16 intratype variants are preferentially associated with specific histological types of cancer [22,86]. However, the information is controversial for HPV-18 variants, since some authors support an association of certain variants with histological types of CC [87,88,89], while others refute it [15].

Knowing the distribution of high-risk HPV intratype variants in different populations, particularly of HPV-16 and HPV-18, will help to identify their risk of developing cervical cancer. A study by Mirabello et al. (2016) included cervical smears from 3200 HPV-16 positive women who routinely attended a clinical center in the US [22]. In this study, the association of HPV variant lineages with precancer and cancer was analyzed. The authors found that 87.2% of the women were infected with an HPV-16 A variant, including European (A1–A3) and Asian (A4) variant sublineages. The presence of A4 variants was significatively associated with an increased risk of CC, compared to A1/A2 (OR = 3.16, 95% CI = 1.05 to 9.54, *p* = 0.04); while women with non-A sublineages (B, C, D) had a higher risk of CIN 3 and CC (OR = 6.87, 95% CI = 4.42–10.68, *p* < 0.0001), which is in agreement with data that non-European variants are associated with a higher risk of developing cancer.

One of the studies with the largest number of samples carried out by Clifford G. et al. (2019) [86], and it analyzed 7116 cervical smears positive for HPV-16 from women from 52 countries. The authors found that the A lineage was the most prevalent, present in 78.7% of the samples, followed by lineages D (9.2%), C (6.4%) and B (5.8%). Regarding the distribution of HPV-16 sublineages, A1 was the most widespread, in Europe, South/Central America, North America, South Asia and Oceania, while sublineages A3 and A4 were mainly prevalent in East Asia. Meanwhile, B and C sublineages were only found in African samples. Increased risk for developing CC was observed for A3, A4 and D sublineages, compared with A1, in regions where those sublineages were more prevalent. When analyzing associations with histological types, sublineage D was more frequent in adenocarcinoma than in squamous cell carcinoma.

All this information supports that even though the distribution of the different variant sublineages of HPV-16 varies widely throughout the world, non-European variants have a higher risk of developing high-grade premalignant lesions and CC.

## 6. Distribution of HPV Variants in Mexico

In Mexico, some studies have been performed to determine HPV intratype variant distribution. An initial work, based on E6 and L1 genes stated that nearly 40% of HPV-16 positive cervical cancer cases in Mexican women belong to Asian–American lineages D2 (AA-c) and D3 (AA-a), which also presented a higher risk of developing cervical cancer than European lineages A1/2 [90], with an OR of 27 and 3.4, respectively. Interestingly, D2 was associated with a younger age of CC development (younger than 50 years old) than D3 [91].

Similar results were found when the HPV-16 E6 gene was sequenced in 40 cases of CC and cervical premalignant lesions from women from the southeast of Mexico (Yucatan) [92]. In this study, the most frequent variants found overall were European-prototype (E-P) (sublineage A1), followed by E-350G (sublineage A2), with no differences among the groups. Furthermore, the most frequent variant in CC was AA (D2), with a 44% rate of positivity in this group, which in turn was not found in premalignant lesions.

Another analysis carried out in 277 samples of premalignant lesions and 133 samples of CC of women who attended primary health care centers and a cancer institute in Mexico City [93] showed that in HPV-16 positive cases, the most common E6 variants were E, followed by AA-a, although no differences were found in their distribution among the groups. When analyzing AA variants together (AA-a plus AA-c), their proportion in CC was up to 41%, similar to that of E variants in this group. Interestingly, although in a low proportion, AA-c (9%) was found exclusively in CC, and not in premalignant lesions, suggesting a more aggressive behavior for this variant.

Subsequently, Escobar Escamilla et al. [94] analyzed the LCR and E6 sequences to study the distribution of HPV-16 lineages in samples with normal cytology, squamous intraepithelial lesions, and CC from 94 patients in Mexico City, the state of Mexico, and the state of Jalisco. Sublineage A was also the most prevalent, representing 82.93% of the samples analyzed, including combined sublineages A1 and A2. The second place in prevalence was occupied by D2 variants (9.75%). Regarding LCR analysis, the most common mutations found were G7191T and G7518A within sublineages A1 and D2, respectively. On the other hand, variant E6-T350G (E-350G) was observed in 80.48% of the cases, where 100% of the CC samples contained this variant. Interestingly, in asymptomatic women in the state of Sinaloa (northwestern Mexico), the E-350G variant has been reported as the most prevalent among HPV-16 variants [95]. This variant has also been found frequently in women with other conditions, such as those analyzed in a study that included samples from the oral cavity and cervix of HIV+ women in Mexico City [96], where an HPV prevalence as high as 96.6% in the cervix and 92.5% in the oral cavity, mainly with high-risk HPV types was found. In 52 of the 174 women from this group that were HPV-16 positive, the most frequent E6 intratype variant was E-350G, being present in both anatomical sites in 80.77% of the cases.

In contrast, in a population of northeastern Mexico (state of Nuevo Leon) where cervical smears of women attending primary health care centers were analyzed, the most common HPV-16 variants found had an AA origin (87%), while European variants were present in 13% of the samples [97]. This confirms that HPV-16 variant distribution varies within the different geographical areas of Mexico.

Other studies analyzed the distribution of HPV-16 E6 and E7 variants from women residents from Guerrero state in southern Mexico and their association with CC and premalignant lesions [98,99]. Analysis of E6 gene variations showed that E-350G was the most prevalent HPV-16 variant in this population (40%); nevertheless, even though AA-a was found in low frequency (10.6%), it presented the highest risk for developing CC (OR = 69.01, CI = 7.57–628.96) [98]. Later, Antaño-Arias et al. (2021) included the E7 gene in the analysis in a total of 190 samples positive to HPV-16 and found 8 mutations within the E7 gene: 2 missense mutations (A647G and C712A) and 6 synonymous mutations (G666A, T678C, T732C, C765T, T789C, and T795G) [99]. In this study, E7 prototype variants, mainly of the A1 and A2 sublineages, were the most prevalent (74.74%), followed by E7-C732/C789/G795 variants (20.53%) (mainly grouped in the D2 sublineage). Similar results were obtained when evaluating the E6 gene in CC, where AA-c and AA-a variants exhibited prevalences of 8.9% and 11.1%, respectively. In cervical tissue without lesions, the most common variants in the E6 gene contained the E-350G mutation. When considering E6/E7 bicistronic variants in relation to the risk of developing cervical cancer and precursor lesions, it was observed that the risk for CC was highest in individuals harboring variant AA-a*E7-C732/C789/G795 (sublineage D2) (OR = 110, 95% CI = 6.04–2001.3, *p* = 0.001), followed by AA-c*E7-C732/C789/G795 (OR = 35, 95% CI = 2.63–465.37).

Little information is available regarding the distribution of HPV-18 variants in Mexico and their association with the risk of developing CC. In a study including 33 cases of premalignant lesions and CC that were positive for HPV-18, significant data was obtained in this regard. The HPV-18 Af variant predominated in normal cytologies and premalignant lesions, while the E variant was exclusively found in CC, being present in 62% of CC cases, which suggests a higher oncogenicity for this E variant [93].

Moreover, cervical swab analysis from a cohort of women attending primary health care centers in Monterrey, Nuevo Leon, showed that in 15 cases positive to HPV-18 the most frequent variants were European, with 13 cases, while 2 cases corresponded to the African variant; however, no information is available regarding HPV-18 variants and cancer risk in this population [97].

It’s worth mentioning that intratype variants of HPV types 31, 35, 62, and 58 have been also characterized from cervical samples of Mexican women from different states of Mexico [97,100,101]. Additionally, variations in HPV-51 have been analyzed in HIV positive Mexican patients, this viral type being highly prevalent in this group [102]. Although no significant associations with the severity of the lesions have yet been found, these studies set precedents to continue analyzing intratype variations of different types of HPV in relation to their distribution and pathogenicity.

The most prevalent variants of HPV-16 and -18 found in the Mexican population are summarized in Table 1.

## 7. Functional Analysis of HPV Variants: From Non-Coding Region Activity to Viral Protein Products, an Interesting Approach in HPV Research in Mexico

Until now, many research groups have focused on functional studies of intratype variants, mainly on the most prevalent types found in the Mexican population. These studies focus on elucidating the mechanisms by which these variants participate in cancer progression and the establishment of persistent infections. Assays that assess transcriptional and replication activity in the LCR, as well as early proteins, such as E6, E7, E1, and E2, are essential to understand the outcome of HPV-related cancers. Notably, most of the performed functional studies focus on deciphering the involvement of E6 variants in processes related to cancer, probably because among HPV oncogenes, E6 harbors more nucleotide variations compared to E7 (Supplementary File).

### 7.1. Long Control Region

**Figure 1:**
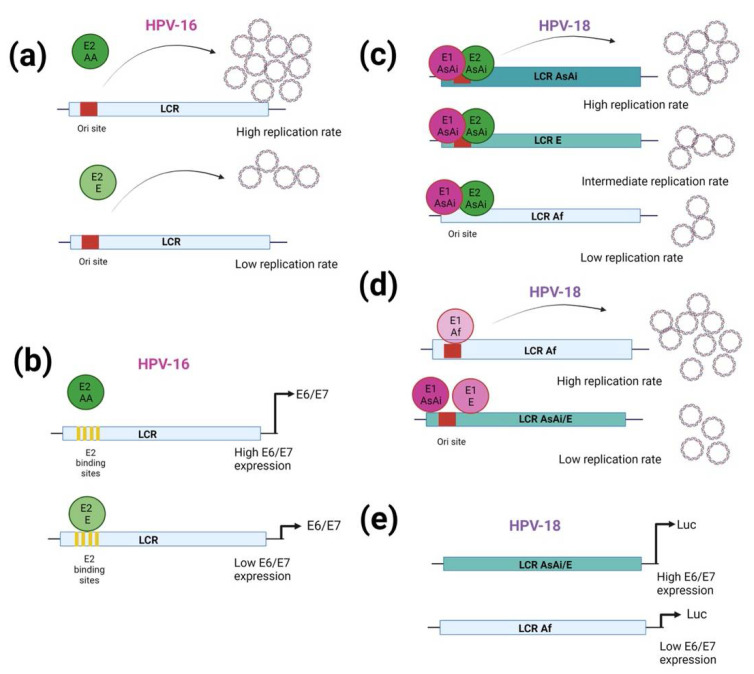
Regulation of the Long Control Region activity by HPV variations. (**a**) HPV-16 positive tumors with E2 AA contain a higher number of viral copies per cell and (**b**) higher E6/E7 transcript levels, in relation to those with E2 E. (**c**) In the presence of HPV18 E1- and E2 AsAi, the Ori site of the AsAi variant exhibit a higher replication rate compared to the Ori sites of E and Af variants. (**d**) When E1 and Ori sites came from the same variant, E1 Af induced the highest Ori viral replication compared to E1 E and AsAi variants, while variations in E2 had no effect. (**e**) Through Luc-reporter plasmid assays, it was shown that HPV-18 LCR Af has a lower transcriptional activity, compared to LCR AsAi or E, suggesting an impact in E6/E7 expression. Figure created in BioRender.

Epidemiological studies reveal that AA HPV-16 variants are more oncogenic than E variants, and such oncogenicity could be due to E6 and E7 activity, viral replication, or transcription enhancement. It was previously suggested that E2 may be involved in the increased oncogenicity of the AA variants through the regulation of E6/E7 transcription. The presence of intact E1/E2 in cervical tumors was shown to correlate with a higher viral copy number than those with a disruption in the E1/E2 region, where a low viral copy number was observed. Moreover, in tumors positive for HPV-16, it was shown that those containing E2 AA (170 ± 38.9) had a higher number of viral copies per cell than those positive for E2 E (67.6 ± 13.5) [103] (Figure 1a). Furthermore, it was suggested that the E6/E7 overexpression is a late event in cases harboring the E variant, and that E6/E7 up-regulation depends on HPV episome integration into the host genome causing the E2 gene to be disrupted [104]. In contrast, carcinomas harboring AA variants retain the E2 gene, suggesting an alternative mechanism for the regulation of E6/E7 transcription. Interestingly, it was observed that in tumors containing AA variants, the levels of E6/E7 transcripts are 2.1 times higher than those with E variants, and that no differences in the status of the viral genome were found between them (Figure 1b). Moreover, the E2 E variant highly repressed the LCR transcriptional activity compared to E2 AA-a and AA-c, and such effect was unrelated to the capacity of E2 to induce apoptosis [104].

In addition to containing E2 binding sites that regulate E6/E7 expression, the LCR also harbors an origin of replication (Ori site), important for the amplification of the viral genome. Another study revealed that variations in HPV-18 E1 and in the LCR differentially affect viral replication. The Ori site of the AsAi variant was found to exhibit a higher replication rate than the Ori site of the E and Af variants, when the E1 and the E2 of AsAi were present (Figure 1c). Furthermore, variations in E2 were shown not to affect Ori function. When evaluating variant homologous elements, that is, when the E1 and Ori sites came from the same variant, E1 Af induced the highest Ori viral replication compared to E1 E and AsAi variants, and such activity was associated with an increased nuclear localization of E1 Af (Figure 1d) [105].

Previously, it was reported that the LCR of the HPV-18 Af variant contains the highest number of nucleotide changes compared to E and AsAi variants, especially in the tissue enhancer region, impacting low transcriptional activity, probably affecting the binding of transcription factors, including KRF-1/OCT1 and OCT-1/TEF1 (Figure 1e). Interestingly, no differences in transcriptional activity were found between LCR AsAi and LCR E variants. Even though several LCR nucleotide changes are found in the HPV-18 variants, it was demonstrated that E2 binding in the LCR did not differ, and E2 repressor activity was unaffected [106].

### 7.2. E6

One way to characterize the specific involvement of high-risk HPV E6 intratype variants is through bioinformatic analyses of gene expression profiles that may ultimately impact in the modulation of cellular processes and cell signaling pathways associated with carcinogenesis.

**Figure 2:**
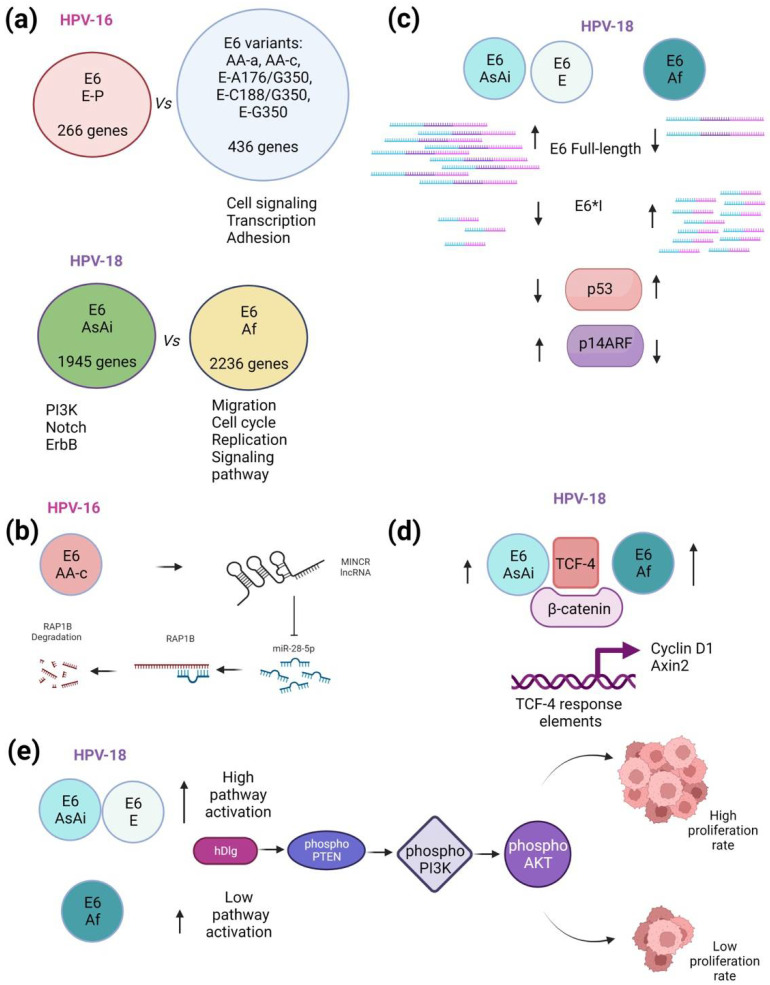
Functional analysis of HPV E6 variants. (**a**) Gene expression profiles of HPV-16 and -18 E6 variants expressing cells. HPV-16 E6 E-P regulates the expression of 266 genes, while the other E6 variants together induced 436 genes, mainly involved in cell signaling, transcription, and adhesion. Furthermore, E6 AsAi and Af from HPV-18 induce the expression of 1945 genes and 2236 genes, respectively, affecting different cellular processes. (**b**) E6 AA-c increases the expression of MINCR, a competitive endogenous RNA of miR-28-5p, preventing the degradation of RAP1B transcript. (**c**) HPV-18 E6 AsAi and E variants exhibit different E6 splicing patterns, being E6 full-length the most prevalent isoform, in contrast E6 Af showed a higher proportion of the small-spliced isoform E6*I, impacting p53 and p14ARF protein levels. (**d**) HPV-18 E6 Af variant induces higher TCF-4 transcriptional activity than E6 AsAi. Both variants bind to TCF-4/β-catenin and induce Cyclin D1 and Axin2 expression. (**e**) HPV-18 E6 AsAi and E variants promote overactivation of the PI3K/AKT pathway compared to E6 Af, differentially impacting proliferation. When indicated, up arrows mean augmented levels, where enlarged arrows refer to higher levels than the short arrows. Down arrows indicate decreased levels. Figure created in BioRender.

Expression patterns were assessed in C-33 A cells stably transfected with HPV-16 E6 E-P, AA-a, AA-c, E-A176/G350, E-C188/G350, and E-G350. It was found that AA-c and E-G350 variants induced the most dramatic changes in transcription, affecting 229 and 132 genes, respectively. Additionally, 436 genes were differentially affected by E6 non-E-P variants in relation to E-P. When biological processes affected by gene expression patterns were analyzed, it was demonstrated that E6 non-E-P variants mainly regulated cell signaling (20.3%), transcription (16.7%), and adhesion (14.6%) (Figure 2a). Particularly, E6 AA-c, E-C188/G350, and E-G350 variants affected processes, including angiogenesis, cell junctions, migration, immune response, apoptosis, cell division, proliferation, and cell signaling pathways [107].

To determine the genome wide expression modulated by E6 variants of HPV-18 found in the Mexican population, an analysis of expression patterns in HaCaT immortalized cells transiently transfected with HPV-18 E6 AsAi and Af variants was carried out. It was found that E6 Af regulates the expression of 2236 genes, while the E6 AsAi variant affected the expression of 1945 genes, and both variants regulated 414 genes in common, suggesting that the E6 HPV protein regulates cellular transcriptional programs regardless of the variant in question. As expected, both variants were able to alter genes related with cancer pathways, E6 AsAi preferentially altered elements of PI3K, Notch, and ErbB signaling, while E6 Af altered processes, such as cell migration, cell cycle, DNA replication, and mTOR signaling and Wnt signaling pathways. In cell proliferation assays, it was observed that E6 AsAi had a greater increase in proliferation than E6 Af. Conversely, migration was higher in cells harboring E6 Af than those with AsAi [108].

Another study showed that HPV-16 E6 variants E-P, AA-c, E-G350, and E-C188/G350 differentially regulate the expression of long non-coding RNAs, including MALAT1, FLVCR1-AS1, CASC15, ZNF667-AS1, SNHG8, CASC19, and MINCR. Interestingly, it was found that MINCR was overexpressed (8-fold) in C-33 A cells harboring E6 AA-c in comparison with the control group. Through bioinformatic analysis, it was found that MINCR is a competitive endogenous RNA (ceRNA) of miR-28-5p, and that RAP1B transcript is a target of miR-28-5p. Furthermore, using data from TCGA of HPV-16 positive cervical cancer cases, it was demonstrated that RAP1B expression was higher, compared to HPV negative cases (Figure 2b); however, no differences were found when evaluating miR-28-5p expression. Moreover, C-33 A cells expressing the E6 AA-c variant exhibited a 0.3-fold decrease expression of miR-28-5p compared to the control group, while the expression and the protein levels of RAP1B increased 21.8- and 2.3-fold, respectively. These data demonstrated an inverse correlation of miR-28-5p and RAP1B in E6 AA-c expressing cells. Additionally, when MINCR was knocked-down in these cells, miR-18-5p expression recovered by 90%, while RAP1B expression was reduced by 60%. Finally, when MINCR was ablated, a reduction of 50% in migration was observed in C-33 A cells harboring HPV-16 E6 AA-c variant. These data are consistent with those previously reported that Asian-American HPV-16 variants exhibit a greater oncogenic potential compared to European variants [109].

Using proteomic analysis, it was demonstrated that E-P, E-G350, E-A176/G350, E-C188/G350, AA-a, and AA-c HPV-16 E6 variants differentially regulate proteins mainly involved in metabolism, including KCNH1, PPP2R1A, CCT8, ALDH1A1, ZN554, ENOA, G6PD, ARMX1, GRHPR, CPT1A, CENP, and ZNF93. In addition, qPCR assays showed that E-A176/G350 and AA-a variants repressed ALDHA1 compared to C-33 A cells transfected with the empty vector, while CCT8 expression was diminished in cells containing E6 E-A176/G350, E-P, and AA-a [110]. Currently, the structure of E6 proteins and their variants has not been characterized under native experimental conditions, which is a limitation to study the interactions with cellular proteins involved in the establishment of cancer. A recent study revealed that variations in E6 genes affect the thermodynamics and structural stability of the 3D protein structure. Through Molecular Dynamics (MD) simulations and docking analyses, it was found that E6 variants, including E-P (reference), E-G350 (L83V), E-C188/G350 (E29Q/L83V), E-A176/G350 (D25N/L83V), AA-a (D25N/L83V), and AA-c (Q14H/I27R/H78Y/L83V) interact with a high affinity with the PDZ-1 domain within MAGI-1. Interestingly, it was observed that E6 E-C188/G350, AA-a, and AA-c proteins showed a greater difference in the C-terminal region in comparison to the E-P variant, while E-G350 and E-A176/G350 maintain similar 3D structures to the E-P variant. Moreover, it was found that residues (141–151) in the C-terminal region of E-P are less flexible than the other tested variants, probably affecting the capacity of the PBM motif (ETQV) to interact with PDZ-containing proteins such MAGI-1. It was found that the E6 non-E-P variants required less energy to interact with MAGI-1, having more affinity for MAGI-1 than the E-P variant, particularly E-A176/G350. The residues responsible for the E6-MAGI-1 interaction were found to be close to the changed nucleotides in the variants, in addition to the fact that the interacting residues differ among the variants analyzed. The detailed study of the interactions that occur between E6 and cellular proteins will allow us to understand the differential processes modulated by E6 variants during the replicative cycle and the establishment of cancer [111].

MCF-7 and C-33 A cells stably expressing AsAi, E, and Af variants were used to investigate the specific contribution of HPV-18 E6 variants. E6 splicing patterns were shown to be different among the tested variants. Cells expressing E6 AsAi and E6-E exhibited high levels of full-length E6 transcript with low levels of E6*I transcript. In contrast, E6 Af produced high levels of E6*I with low amounts of full-length E6 (Figure 2c). Interestingly, these expression patterns were similar in tumors containing HPV-18 variants [18]. The E6 mRNA profile of the tested variants was in concordance with E6 protein patterns. The reduction of functional full-length E6 may partly explain the biological behavior observed in HPV-18 premalignant lesions and cancer. Interestingly, p53 levels decreased in the presence of E6 AsAi and E, while in cells harboring E6 Af, p53 levels were unchanged (Figure 2c). An in vitro p53 degradation assay was performed to evaluate the specific effect of variations in full-length E6, where no splicing should occur; surprisingly, no differences were found between E6 AsAi and Af variants in their ability to induce p53 degradation, confirming that the reduced proportion of full-length E6 in the African variant impacts the reduction of p53 degradation. Additionally, biological assays revealed that cells with AsAi and E variants were able to induce colony formation and tumor growth more quickly than the E6 Af variant. Interestingly, when the E6 Af variant was mutated (C491A), a change in E6 expression pattern was obtained that was similar to that observed for the E6 AsAi variant, and even the capacity to induce p53 degradation was restored. Taken together, these data suggest that differences in oncogenic capacity displayed by HPV-18 E6 variants are highly dependent on their splicing patterns [18].

In agreement with these data, using MCF-7 cells stably transfected with HPV-18 E6 AsAi, E, and Af variants, differences in E6/E6*I ratio among variants were confirmed, showing that E6 Af produced a higher proportion of the E6*I transcript. Subsequently, p53 protein levels and its downstream effector p14ARF were evaluated in the presence of E6 variants. As expected, p53 protein was ablated in E6 AsAi and E expressing cells, whereas a low reduction of p53 levels was observed in cells with E6 Af. In contrast, p14ARF, a gene negatively regulated by p53, was augmented in the presence of E6 AsAi and E variants and decreased in cells with E6 Af (Figure 2c). Those effects were attributed to a higher proportion of E6*I and low full-length E6 levels in cells with E6 Af, where E6 is limited in its ability to degrade p53 [112].

HPV-18 E6 variants were shown to differentially regulate the activation of cell signaling pathways involved in proliferation, such as Wnt/β-catenin and PI3K/Akt. In C-33 A cells transiently transfected with the E6 AsAi variant, a 1.5-fold increase in TCF-4 transcriptional activity was observed compared to those cells transfected with the empty vector. Interestingly, the E6 Af variant induced a significant increase (2.5-fold) in TCF-4 transcriptional activity above that seen with the E6 AsAi variant (Figure 2d). Such activity was enhanced in E6 AsAi and Af expressing cells when β-catenin was exogenously overexpressed (1.6- and 2.25-fold above control, respectively). Cyclin D1 and Axin2 genes were similarly up-regulated in the presence of E6 AsAi and Af variants. E6 variants were found to interact with β-catenin and TCF-4. Additionally, TCF-4 stability and nuclear levels were increased in the presence of E6 AsAi; and, surprisingly, E6 AsAi was also found to bind to the SP5 promoter, which is transcriptionally regulated by TCF-4. Finally, the contribution of E6 AsAi in the regulation of the Wnt/β-catenin pathway was to increase cell proliferation [113].

The effect of HPV-18 E6 variations on the PI3K/Akt pathway was evaluated. Protein levels of PTEN, an inhibitor of PI3K kinase, were found to remain unchanged in MCF-7 and C-33 A cells expressing E6 AsAi, E6 E variants, or without E6. However, when phospho-PTEN was assessed, virtually no phospho-PTEN was found in cells with E6 AsAi or E6 E, while reduced phospho-PTEN levels were found in cells expressing E6 Af compared to cells with no E6 (Figure 2e). Additionally, hDlg, a negative PTEN regulator, was reduced in the presence of E6 variants, although E6 Af produced a smaller reduction of hDlg levels relative to E and AsAi variants. In addition, the PTEN/hDlg interaction was decreased in cells expressing the Af variant. A similar effect occurred in the levels of phospho-Akt/PKB, phospho-PI3K, and phospho-ERK1/2, since the presence of E6 AsAi and E variants increased those levels, suggesting a strong activation of the pathway, while the Af variant induced a modest activation of Akt/PKB and no activation of ERK1/2 or PI3K. Consistent with these results, cells expressing E6 AsAi and E6-E increased cell proliferation, while the lowest proliferation rate was observed for cells expressing E6 Af. As described above, the splicing patterns in E6 transcripts differ among E6 HPV-18 variants. Therefore, those differences in phospho-PTEN levels observed among AsAi and E variants compared to Af, could be attributable to the fact that E6 AsAi and E retained the full-length E6 isoform, while E6 Af produced practically only the E6*I transcript [114]. It is likely that the effect observed on cell proliferation by each of the variants is partially due to an effect exerted by changes in the proportions of E6 isoforms. In this sense, different proportions of E6 and E6* isoforms are found depending on various cellular stimuli. It has been reported that both E6 and E6* exhibit controversial functions, for example, E6 induces p53 degradation, while E6* interferes with E6-dependent p53 degradation. Furthermore, unlike E6, E6* does not induce keratinocyte immortalization, but instead reduces cell proliferation and the tumor size of HPV-16 positive cancer cells. Additionally, E6 and E6*I have been shown to increase the levels and the activity of caspase 8, in addition to inducing its nuclear translocation, however, they do not promote apoptosis [115].

### 7.3. E7

To elucidate the specific participation of the intratype variants of E7 in cancer processes, one in silico study has been carried out to establish differences in the three-dimensional structure of HPV-16 E7 N29S and H51N variants in comparison with the E-prototype. Those variants have been most frequently associated with cervical cancer. The amino acid changes within variants are located in CR2 and CR3 domains for N29S and H51N, respectively. Interestingly, it was reported that the change of N29S is susceptible for phosphorylation that may reinforce nearby sites (S31 and S32) also susceptible to phosphorylation which could affect the structure of E7. Molecular dynamics (MD) analysis revealed that the N29S variant exhibits a more compact structure than the E7 E-P and H51N variants. Furthermore, 3D structure was evaluated comparing E7 variants, revealing major changes in the CR2 and CR3 domains that may affect the interaction with different cellular proteins, including pRb. Further, when analyzing the 2D structure by MD, it was demonstrated that E7 E-P lost its α-helix and β-sheets, while the H51N variant conserved the α-helix but lost the β-sheets, while most of the secondary structures of N29S were preserved. These results indicate that N29S variant is more structurally stable than the reference E7 and H51N. Finally, using analysis of electrostatic potential, differences in the charge on the LXCXE motif were also observed. The E7 E-P variant essentially presents a negative charge in this region, while the N29S variant harbors a combination of negative, electrostatically positive, and neutral charges in this region. Meanwhile, the H51N variant contains negative, neutral, and positive regions in the LXCXE motif. These interesting findings suggest that the affinity of cellular proteins for E7, which is dependent on the LXCXE motif may differ between variants, which may affect their oncogenic potential [116].

### 7.4. E2

In relation to the participation of E2 variants in cancer, hitherto only one functional study has been carried out. In order to have a more comprehensive idea of the participation of E2 AsAi and-Af variants of HPV-18 in processes associated with cancer, the transcriptional profiles altered by E2 and its variants were assessed in different cell lines, including MCF-7, C-33 A, and HEK293. The analyses included the results of the three cell lines in order to evaluate the effect of the E2 variants regardless of the cellular context, that is, the aim was to show the genes that were exclusively deregulated by the E2 variants regardless of the cell type. It was demonstrated that E2 variants differentially regulated gene transcription patterns where expression of 1361 genes were affected by E2 AsAi, while E2 Af altered the expression of 235 genes, and only 34 genes were shared between the E2 tested variants. Interestingly, gene ontology analysis demonstrated that both AsAi and Af variants regulated cellular processes, including metabolism (523 vs. 73 genes), proliferation (78 vs. 15 genes), cellular communication (252 vs. 59 genes), and death (93 vs. 18 genes), respectively. It was demonstrated that the effect of E2 variants on gene regulation was maintained in different cell contexts. Moreover, it was previously demonstrated that E2 induced cell death; however, when comparing the effects of E2 variants it was found that E2 AsAi favors gene expression related to the extrinsic apoptotic pathway, while E2 Af favors the intrinsic apoptotic pathway. Through viability assays, it was shown that both variants reduced cell viability in comparison to control cells (77.63% and 46.27% for E2 AsAi and E2 Af, respectively); however, the strongest effect was observed in those cells with E2 Af. Similar results were obtained when apoptosis was evaluated, it was found that both variants differentially induced apoptosis in relation to the control cells, being 29.4% attributable to E2 AsAi, while E2 Af induced 53.3%. These results suggest that E2 variants differentially regulate cellular viability and apoptosis, probably due to the different gene expression profiles induced by the variants [117].

## 8. Concluding Remarks: Where We Are and Where We Are Going?

Cervical cancer continues to be a serious public health problem in Mexico, since mortality related to this type of cancer has had a significant increase from 3357 cases reported in 2012 to 4335 cases in 2020 [118]. The vaccination program in Mexico was introduced in 2012 for 11-year-old girls, who have not started their sexual life, and it includes two doses of the quadrivalent vaccine, where the second dose is applied at six months up to one year after the first dose. According to official reports provided by the World Health Organization portal, a coverage of more than 80% of vaccination against papillomavirus has been achieved in Mexico in this population, during the period 2012–2019. Notably, these numbers decreased drastically in 2020 to 11.22% and, unfortunately, 0.45% was reported for 2021, probably due to the health policy in our country being mainly focused on dealing with the COVID pandemic [119]. To our knowledge, there are so far no studies available that evaluate vaccination coverage in addition to highlighting the efficacy of papillomavirus vaccines in Mexico. Unfortunately, the incidence and the mortality of cervical cancer and other HPV-related cancers continue to rise. In addition, the effect of immunization against papillomavirus granted by vaccination programs in the prevention of cervical cancer could be reflected in the coming years (more than 20 years), when the first vaccinated population reaches the age at which the greatest number of cases of cervical cancer currently occur. Likewise, contemplating the beginning of the vaccination scheme in young men will allow for controlling the spread of papillomavirus infection, in addition to encompassing the prevention of the development of other HPV-related tumors, including penile, anus, and oropharynx cancers [120,121,122]. The participation of various research groups throughout Mexico, has provided cutting-edge knowledge in the study of papillomavirus and cancer, either through epidemiological or functional studies of HR-HPV and its variants, which allow for defining the geographical distribution of HPVs and understanding the molecular and the cellular events affected by these viruses. Interestingly, as highlighted in the previous sections, HPV-16 is the most prevalent viral type cancer in the Mexican population, and even more interesting is that some intratype variants are commonly found in cancer samples that confer risk of progression to develop cancer. This suggests that variants of the same viral type differentially and finely regulate cellular processes that ultimately lead to cancer. Furthermore, it is of interest to observe the effect of vaccines on the protection against tumors that contain the same HPV type but have a different variant. Understanding the biological behavior of intratype variants of papillomaviruses associated with cancer will eventually allow for the development of targeted strategies aimed not only at preventing cervical cancer but also at predicting the outcome of patients currently suffering from this type of cancer.

## Figures and Tables

**Table 1:** HPV-16 and -18 variants commonly found in cancer among Mexican patients.

Region	HPV	HPV Gene	Variant/Sublineage	Prevalence in Cancer	Cancer Risk (OR)	Reference
Mexico City	16	E6/L1	D2 (AA-c)	23.2%	27	[90]
			D3 (AA-a)			
			European	27.1%	3.4	
						
Mexico City	16	E6/L1/LCR	A1/A2	31.2%	1.3	[91]
			D2	10.8%	3.3	
			D3	8.9%	0.6	
						
Mexico City	16	E6	European	58%	-	[93]
			D3 (AA-a)	32.3%	-	
			D2 (AA-c)	8.8%	-	
						
Mexico City	16	E6	E350G	80.77%	-	[96]
						
Mexico City, Mexico State, Jalisco	16	E6/LCR	A1/A2	82%	-	[94]
						
Mexico City	18	E6/LCR	European	62%	-	[93]
			Af	24%	-	
			AsAi	14%	-	
						
Guerrero	16	E6	E350G	40%	-	[98]
			AA-a	10.6%	69	
						
Guerrero	16	E7	E7-prototype (A1, A2)	74.74%	1	[99]
			E7-C732/C789/G795 (D2)	20.53%	3.79	
		E6/E7	E6-AA-a/E7- C732/C789/G795 (D2)	11.1%	110	
			E6-AA-c/E7- C732/C789/G795 (D2)	8.9%	35	
						
Yucatan	16	E6	D2 (AA)	44%	-	[92]

## Supplementary Materials

The supporting information can be downloaded at: https://www.mdpi.com/article/10.3390/ijms23158566/s1.

## References

[1] de Martel C., Georges D., Bray F., Ferlay J., Clifford G.M. (2020). Global Burden of Cancer Attributable to Infections in 2018: A Worldwide Incidence Analysis. Lancet Glob. Health.

[2] de Sanjose S., Quint W.G.V., Alemany L., Geraets D.T., Klaustermeier J.E., Lloveras B., Tous S., Felix A., Bravo L.E., Shin H.R. (2010). Human Papillomavirus Genotype Attribution in Invasive Cervical Cancer: A Retrospective Cross-Sectional Worldwide Study. Lancet Oncol..

[3] Adebamowo S.N., Befano B., Cheung L.C., Rodriguez A.C., Demarco M., Rydzak G., Chen X., Porras C., Herrero R., Kim J.J. (2022). Different Human Papillomavirus Types Share Early Natural History Transitions in Immunocompetent Women. Int. J. Cancer.

[4] Plummer M., de Martel C., Vignat J., Ferlay J., Bray F., Franceschi S. (2016). Global Burden of Cancers Attributable to Infections in 2012: A Synthetic Analysis. Lancet Glob. Health.

[5] de Martel C., Plummer M., Vignat J., Franceschi S. (2017). Worldwide Burden of Cancer Attributable to HPV by Site, Country and HPV Type. Int. J. Cancer.

[6] Kombe A.J., Li B., Zahid A., Mengist H.M., Bounda G.A., Zhou Y., Jin T. (2021). Epidemiology and Burden of Human Papillomavirus and Related Diseases, Molecular Pathogenesis, and Vaccine Evaluation. Front. Public Health.

[7] Sung H., Ferlay J., Siegel R.L., Laversanne M., Soerjomataram I., Jemal A., Bray F. (2021). Global Cancer Statistics 2020: GLOBOCAN Estimates of Incidence and Mortality Worldwide for 36 Cancers in 185 Countries. CA A Cancer J. Clin..

[8] Bzhalava D., Guan P., Franceschi S., Dillner J., Clifford G. (2013). A Systematic Review of the Prevalence of Mucosal and Cutaneous Human Papillomavirus Types. Virology.

[9] Bernard H.U., Burk R.D., Chen Z., van Doorslaer K., Hausen H.Z., de Villiers E.M. (2010). Classification of Papillomaviruses (PVs) Based on 189 PV Types and Proposal of Taxonomic Amendments. Virology.

[10] Van Doorslaer K., Li Z., Xirasagar S., Maes P., Kaminsky D., Liou D., Sun Q., Kaur R., Huyen Y., McBride A.A. (2017). The Papillomavirus Episteme: A Major Update to the Papillomavirus Sequence Database. Nucleic Acids Res..

[11] Guan P., Howell-Jones R., Li N., Bruni L., De Sanjosé S., Franceschi S., Clifford G.M. (2012). Human Papillomavirus Types in 115,789 HPV-Positive Women: A Meta-Analysis from Cervical Infection to Cancer. Int. J. Cancer.

[12] LeConte B.A., Szaniszlo P., Fennewald S.M., Lou D.I., Qiu S., Chen N.W., Lee J.H., Resto V.A. (2018). Differences in the Viral Genome between HPV-Positive Cervical and Oropharyngeal Cancer. PLoS ONE.

[13] Serrano B., De Sanjosé S., Tous S., Quiros B., Muñoz N., Bosch X., Alemany L. (2015). Human Papillomavirus Genotype Attribution for HPVs 6, 11, 16, 18, 31, 33, 45, 52 and 58 in Female Anogenital Lesions. Eur. J. Cancer.

[14] Schrank T.P., Landess L., Stepp W.H., Rehmani H., Weir W.H., Lenze N., Lal A., Wu D., Kothari A., Hackman T.G. (2022). Comprehensive Viral Genotyping Reveals Prognostic Viral Phylogenetic Groups in HPV16-Associated Squamous Cell Carcinoma of the Oropharynx. Mol. Cancer Res..

[15] Chen A.A., Gheit T., Franceschi S., Tommasino M., Clifford G.M. (2015). Human Papillomavirus 18 Genetic Variation and Cervical Cancer Risk Worldwide. J. Virol..

[16] De Villiers E.M., Fauquet C., Broker T.R., Bernard H.U., Zur Hausen H. (2004). Classification of Papillomaviruses. Virology.

[17] Burk R.D., Harari A., Chen Z. (2013). Human Papillomavirus Genome Variants. Virology.

[18] De la Cruz-Hernández E., García-Carrancá A., Mohar-Betancourt A., Dueñas-González A., Contreras-Paredes A., Pérez-Cardenas E., Herrera-Goepfert R., Lizano-Soberón M. (2005). Differential Splicing of E6 within Human Papillomavirus Type 18 Variants and Functional Consequences. J. Gen. Virol..

[19] Hadami K., Saby C., Dakka N., Collin G., Attaleb M., Khyatti M., Filali-Maltouf A., Morjani H., El Mzibri M. (2021). Degradation of P53 by HPV16-E6 Variants Isolated from Cervical Cancer Specimens of Moroccan Women. Gene.

[20] Zhao J., Zhu J., Guo J., Zhu T., Zhong J., Liu M., Ruan Y., Liao S., Li F. (2020). Genetic Variability and Functional Implication of HPV16 from Cervical Intraepithelial Neoplasia in Shanghai Women. J. Med. Virol..

[21] Hochmann J., Sobrinho J.S., Villa L.L., Sichero L. (2016). The Asian-American Variant of Human Papillomavirus Type 16 Exhibits Higher Activation of MAPK and PI3K/AKT Signaling Pathways, Transformation, Migration and Invasion of Primary Human Keratinocytes. Virology.

[22] Mirabello L., Yeager M., Cullen M., Boland J.F., Chen Z., Wentzensen N., Zhang X., Yu K., Yang Q., Mitchell J. (2016). HPV16 Sublineage Associations with Histology-Specific Cancer Risk Using HPV Whole-Genome Sequences in 3200 Women. J. Natl. Cancer Inst..

[23] Cornet I., Gheit T., Iannacone M.R., Vignat J., Sylla B.S., Del Mistro A., Franceschi S., Tommasino M., Clifford G.M. (2013). HPV16 Genetic Variation and the Development of Cervical Cancer Worldwide. Br. J. Cancer.

[24] Gheit T., Cornet I., Clifford G.M., Iftner T., Munk C., Tommasino M., Kjaer S.K. (2011). Risks for Persistence and Progression by Human Papillomavirus Type 16 Variant Lineages among a Population-Based Sample of Danish Women. Cancer Epidemiol. Biomark. Prev..

[25] Schiffman M., Rodriguez A.C., Chen Z., Wacholder S., Herrero R., Hildesheim A., Desalle R., Befano B., Yu K., Safaeian M. (2010). A Population-Based Prospective Study of Carcinogenic Human Papillomavirus Variant Lineages, Viral Persistence, and Cervical Neoplasia. Cancer Res..

[26] Sichero L., Ferreira S., Trottier H., Duarte-Franco E., Ferenczy A., Franco E.L., Villa L.L. (2007). High Grade Cervical Lesions Are Caused Preferentially by Non-European Variants of HPVs 16 and 18. Int. J. Cancer.

[27] Freitas L.B., Chen Z., Muqui E.F., Boldrini N.A.T., Miranda A.E., Spano L.C., Burk R.D. (2014). Human Papillomavirus 16 Non-European Variants Are Preferentially Associated with High-Grade Cervical Lesions. PLoS ONE.

[28] Mane A., Limaye S., Patil L., Kulkarni-Kale U. (2022). Genetic Variations in the Long Control Region of Human Papillomavirus Type 16 Isolates from India: Implications for Cervical Carcinogenesis. J. Med. Microbiol..

[29] Larijani M.S., Omrani M.D., Soleimani R., Bavand A., Nejadeh A.H., Ezzatizadeh V., Jamshidi M., Ramezani A. (2022). Determination of Human Papillomavirus Type 18 Lineage of E6: A Population Study from Iran. Biomed. Res. Int..

[30] Zheng Z.M., Baker C.C. (2006). Papillomavirus Genome Structure, Expression, and Post-Transcriptional Regulation. Front. Biosci. A J. Virtual Libr..

[31] Ozbun M.A., Campos S.K. (2021). The Long and Winding Road: Human Papillomavirus Entry and Subcellular Trafficking. Curr. Opin. Virol..

[32] Schelhaas M., Shah B., Holzer M., Blattmann P., Kühling L., Day P.M., Schiller J.T., Helenius A. (2012). Entry of Human Papillomavirus Type 16 by Actin-Dependent, Clathrin- and Lipid Raft-Independent Endocytosis. PLoS Pathog..

[33] Shafti-Keramat S., Handisurya A., Kriehuber E., Meneguzzi G., Slupetzky K., Kirnbauer R. (2003). Different Heparan Sulfate Proteoglycans Serve as Cellular Receptors for Human Papillomaviruses. J. Virol..

[34] Surviladze Z., Sterk R.T., DeHaro S.A., Ozbun M.A. (2013). Cellular Entry of Human Papillomavirus Type 16 Involves Activation of the Phosphatidylinositol 3-Kinase/Akt/MTOR Pathway and Inhibition of Autophagy. J. Virol..

[35] Evander M., Frazer I.H., Payne E., Qi Y.M., Hengst K., McMillan N.A. (1997). Identification of the Alpha6 Integrin as a Candidate Receptor for Papillomaviruses. J. Virol..

[36] Spoden G., Freitag K., Husmann M., Boller K., Sapp M., Lambert C., Florin L. (2008). Clathrin- and Caveolin-Independent Entry of Human Papillomavirus Type 16—Involvement of Tetraspanin-Enriched Microdomains (TEMs). PLoS ONE.

[37] Finke J., Hitschler L., Boller K., Florin L., Lang T. (2020). HPV Caught in the Tetraspanin Web?. Med. Microbiol. Immunol..

[38] Dziduszko A., Ozbun M.A. (2013). Annexin A2 and S100A10 Regulate Human Papillomavirus Type 16 Entry and Intracellular Trafficking in Human Keratinocytes. J. Virol..

[39] Cerqueira C., Ventayol P.S., Vogeley C., Schelhaas M. (2015). Kallikrein-8 Proteolytically Processes Human Papillomaviruses in the Extracellular Space to Facilitate Entry into Host Cells. J. Virol..

[40] Richards R.M., Lowy D.R., Schiller J.T., Day P.M. (2006). Cleavage of the Papillomavirus Minor Capsid Protein, L2, at a Furin Consensus Site Is Necessary for Infection. Proc. Natl. Acad. Sci. USA.

[41] Inoue T., Zhang P., Zhang W., Goodner-Bingham K., Dupzyk A., DiMaio D., Tsai B. (2018). γ-Secretase Promotes Membrane Insertion of the Human Papillomavirus L2 Capsid Protein during Virus Infection. J. Cell Biol..

[42] Harwood M.C., Dupzyk A.J., Inoue T., DiMaio D., Tsai B. (2020). P120 Catenin Recruits HPV to γ-Secretase to Promote Virus Infection. PLoS Pathog..

[43] Bergant Marušič M., Ozbun M.A., Campos S.K., Myers M.P., Banks L. (2012). Human Papillomavirus L2 Facilitates Viral Escape from Late Endosomes via Sorting Nexin 17. Traffic.

[44] Lipovsky A., Popa A., Pimienta G., Wyler M., Bhan A., Kuruvilla L., Guie M.A., Poffenberger A.C., Nelson C.D.S., Atwood W.J. (2013). Genome-Wide SiRNA Screen Identifies the Retromer as a Cellular Entry Factor for Human Papillomavirus. Proc. Natl. Acad. Sci. USA.

[45] Di Giuseppe S., Luszczek W., Keiffer T.R., Bienkowska-Haba M., Guion L.G.M., Sapp M.J. (2016). Incoming Human Papillomavirus Type 16 Genome Resides in a Vesicular Compartment throughout Mitosis. Proc. Natl. Acad. Sci. USA.

[46] Calton C.M., Bronnimann M.P., Manson A.R., Li S., Chapman J.A., Suarez-Berumen M., Williamson T.R., Molugu S.K., Bernal R.A., Campos S.K. (2017). Translocation of the Papillomavirus L2/VDNA Complex across the Limiting Membrane Requires the Onset of Mitosis. PLoS Pathog..

[47] Aydin I., Villalonga-Planells R., Greune L., Bronnimann M.P., Calton C.M., Becker M., Lai K.Y., Campos S.K., Schmidt M.A., Schelhaas M. (2017). A Central Region in the Minor Capsid Protein of Papillomaviruses Facilitates Viral Genome Tethering and Membrane Penetration for Mitotic Nuclear Entry. PLoS Pathog..

[48] Day P.M., Baker C.C., Lowy D.R., Schiller J.T. (2004). Establishment of Papillomavirus Infection Is Enhanced by Promyelocytic Leukemia Protein (PML) Expression. Proc. Natl. Acad. Sci. USA.

[49] McBride A.A. (2013). The Papillomavirus E2 Proteins. Virology.

[50] Bergvall M., Melendy T., Archambault J. (2013). The E1 Proteins. Virology.

[51] Maglennon G.A., McIntosh P., Doorbar J. (2011). Persistence of Viral DNA in the Epithelial Basal Layer Suggests a Model for Papillomavirus Latency Following Immune Regression. Virology.

[52] Castro-Muñoz L.J., Manzo-Merino J., Muñoz-Bello J.O., Olmedo-Nieva L., Cedro-Tanda A., Alfaro-Ruiz L.A., Hidalgo-Miranda A., Madrid-Marina V., Lizano M. (2019). The Human Papillomavirus (HPV) E1 Protein Regulates the Expression of Cellular Genes Involved in Immune Response. Sci. Rep..

[53] Moody C.A., Laimins L.A. (2010). Human Papillomavirus Oncoproteins: Pathways to Transformation. Nat. Rev. Cancer.

[54] Bello J.O.M., Nieva L.O., Paredes A.C., Gonzalez A.M.F., Zavaleta L.R., Lizano M. (2015). Regulation of the Wnt/β-Catenin Signaling Pathway by Human Papillomavirus E6 and E7 Oncoproteins. Viruses.

[55] Manzo-Merino J., Contreras-Paredes A., Vázquez-Ulloa E., Rocha-Zavaleta L., Fuentes-Gonzalez A.M., Lizano M. (2014). The Role of Signaling Pathways in Cervical Cancer and Molecular Therapeutic Targets. Arch. Med. Res..

[56] Vázquez-Ulloa E., Lizano M., Sjöqvist M., Olmedo-Nieva L., Contreras-Paredes A. (2018). Deregulation of the Notch Pathway as a Common Road in Viral Carcinogenesis. Rev. Med. Virol..

[57] Gutierrez-Xicotencatl L., Pedroza-Saavedra A., Chihu-Amparan L., Salazar-Piña A., Maldonado-Gama M., Esquivel-Guadarrama F. (2021). Cellular Functions of HPV16 E5 Oncoprotein during Oncogenic Transformation. Mol. Cancer Res. MCR.

[58] Doorbar J. (2013). The E4 Protein; Structure, Function and Patterns of Expression. Virology.

[59] McIntosh P.B., Laskey P., Sullivan K., Davy C., Wang Q., Jackson D.J., Griffin H.M., Doorbar J. (2010). E1–E4-Mediated Keratin Phosphorylation and Ubiquitylation: A Mechanism for Keratin Depletion in HPV16-Infected Epithelium. J. Cell Sci..

[60] Buck C.B., Trus B.L. (2012). The Papillomavirus Virion: A Machine Built to Hide Molecular Achilles’ Heels. Adv. Exp. Med. Biol..

[61] Hussain S.S., Lundine D., Leeman J.E., Higginson D.S. (2021). Genomic Signatures in HPV-Associated Tumors. Viruses.

[62] Chesson H.W., Dunne E.F., Hariri S., Markowitz L.E. (2014). The Estimated Lifetime Probability of Acquiring Human Papillomavirus in the United States. Sex. Transm. Dis..

[63] Gravitt P.E. (2011). The Known Unknowns of HPV Natural History. J. Clin. Investig..

[64] Shanmugasundaram S., You J. (2017). Targeting Persistent Human Papillomavirus Infection. Viruses.

[65] Chen H.C., Schiffman M., Lin C.Y., Pan M.H., You S.L., Chuang L.C., Hsieh C.Y., Liaw K.L., Hsing A.W., Chen C.J. (2011). Persistence of Type-Specific Human Papillomavirus Infection and Increased Long-Term Risk of Cervical Cancer. J. Natl. Cancer Inst..

[66] Gheit T. (2019). Mucosal and Cutaneous Human Papillomavirus Infections and Cancer Biology. Front. Oncol..

[67] Graham S.V. (2017). The Human Papillomavirus Replication Cycle, and Its Links to Cancer Progression: A Comprehensive Review. Clin. Sci..

[68] Martin C.M., O’Leary J.J. (2011). Histology of Cervical Intraepithelial Neoplasia and the Role of Biomarkers. Best Pract. Research. Clin. Obstet. Gynaecol..

[69] Ho L., Chan S.Y., Burk R.D., Das B.C., Fujinaga K., Icenogle J.P., Kahn T., Kiviat N., Lancaster W., Mavromara-Nazos P. (1993). The Genetic Drift of Human Papillomavirus Type 16 Is a Means of Reconstructing Prehistoric Viral Spread and the Movement of Ancient Human Populations. J. Virol..

[70] Yamada T., Manos M.M., Peto J., Greer C.E., Munoz N., Bosch F.X., Wheeler C.M. (1997). Human Papillomavirus Type 16 Sequence Variation in Cervical Cancers: A Worldwide Perspective. J. Virol..

[71] Wheeler C.M., Yamada T., Hildesheim A., Jenison S.A. (1997). Human Papillomavirus Type 16 Sequence Variants: Identification by E6 and L1 Lineage-Specific Hybridization. J. Clin. Microbiol..

[72] Cornet I., Gheit T., Franceschi S., Vignat J., Burk R.D., Sylla B.S., Tommasino M., Clifford G.M. (2012). Human Papillomavirus Type 16 Genetic Variants: Phylogeny and Classification Based on E6 and LCR. J. Virol..

[73] Mirabello L., Clarke M.A., Nelson C.W., Dean M., Wentzensen N., Yeager M., Cullen M., Boland J.F., Alemany L., Banks L. (2018). The Intersection of HPV Epidemiology, Genomics and Mechanistic Studies of HPV-Mediated Carcinogenesis. Viruses.

[74] PaVE The Papillomavirus Episteme. https://pave.niaid.nih.gov/.

[75] Asensio-Puig L., Alemany L., Pavón M.A. (2022). A Straightforward HPV16 Lineage Classification Based on Machine Learning. Front. Artif. Intell..

[76] Ong C.K., Chan S.Y., Campo M.S., Fujinaga K., Mavromara-Nazos P., Labropoulou V., Pfister H., Tay S.K., ter Meulen J., Villa L.L. (1993). Evolution of Human Papillomavirus Type 18: An Ancient Phylogenetic Root in Africa and Intratype Diversity Reflect Coevolution with Human Ethnic Groups. J. Virol..

[77] Sichero L., Sobrinho J.S., Villa L.L. (2012). Oncogenic Potential Diverge among Human Papillomavirus Type 16 Natural Variants. Virology.

[78] Ferreira M.T., Gonçalves M.G., López R.V.M., Sichero L. (2021). Genetic Variants of HPV-16 and Their Geographical and Anatomical Distribution in Men: A Systematic Review with Meta-Analysis. Virology.

[79] Cochicho D., da Costa R.G., Felix A. (2021). Exploring the Roles of HPV16 Variants in Head and Neck Squamous Cell Carcinoma: Current Challenges and Opportunities. Virol. J..

[80] Kukimoto I., Muramatsu M. (2015). Genetic Variations of Human Papillomavirus Type 16: Implications for Cervical Carcinogenesis. Jpn. J. Infect. Dis..

[81] Pientong C., Wongwarissara P., Ekalaksananan T., Swangphon P., Kleebkaow P., Kongyingyoes B., Siriaunkgul S., Tungsinmunkong K., Suthipintawong C. (2013). Association of Human Papillomavirus Type 16 Long Control Region Mutation and Cervical Cancer. Virol. J..

[82] Villa L.L., Sichero L., Rahal P., Caballero O., Ferenczy A., Rohan T., Franco E.L. (2000). Molecular Variants of Human Papillomavirus Types 16 and 18 Preferentially Associated with Cervical Neoplasia. J. Gen. Virol..

[83] Totaro M.E., Gili J.A., Liotta D.J., Schurr T.G., Picconi M.A., Badano I. (2022). Genetic Variation in the E6 and E7 Genes of Human Papillomavirus Type 16 in Northeastern Argentina. J. Med. Virol..

[84] Pista A., Oliveira A., Barateiro A., Costa H., Verdasca N., Paixão M.T. (2007). Molecular Variants of Human Papillomavirus Type 16 and 18 and Risk for Cervical Neoplasia in Portugal. J. Med. Virol..

[85] Xi L.F., Koutsky L.A., Hildesheim A., Galloway D.A., Wheeler C.M., Winer R.L., Ho J., Kiviat N.B. (2007). Risk for High-Grade Cervical Intraepithelial Neoplasia Associated with Variants of Human Papillomavirus Types 16 and 18. Cancer Epidemiol. Biomark. Prev. A Publ. Am. Assoc. Cancer Res. Cosponsored Am. Soc. Prev. Oncol..

[86] Clifford G.M., Tenet V., Georges D., Alemany L., Pavón M.A., Chen Z., Yeager M., Cullen M., Boland J.F., Bass S. (2019). Human Papillomavirus 16 Sub-Lineage Dispersal and Cervical Cancer Risk Worldwide: Whole Viral Genome Sequences from 7116 HPV16-Positive Women. Papillomavirus Res..

[87] Lizano M., Berumen J., Guido M.C., Casas L., García-Carranca A. (1997). Association between Human Papillomavirus Type 18 Variants and Histopathology of Cervical Cancer. J. Natl. Cancer Inst..

[88] Burk R.D., Terai M., Gravitt P.E., Brinton L.A., Kurman R.J., Barnes W.A., Greenberg M.D., Hadjimichael O.C., Fu L., Mcgowan L. (2003). Distribution of Human Papillomavirus Types 16 and 18 Variants in Squamous Cell Carcinomas and Adenocarcinomas of the Cervix. Cancer Res..

[89] De Boer M.A., Peters L.A.W., Aziz M.F., Siregar B., Cornain S., Vrede M.A., Jordanova E.S., Fleuren G.J. (2005). Human Papillomavirus Type 18 Variants: Histopathology and E6/E7 Polymorphisms in Three Countries. Int. J. Cancer.

[90] Berumen J., Ordoñez R.M., Lazcano E., Salmeron J., Galvan S.C., Estrada R.A., Yunes E., Garcia-Carranca A., Gonzalez-Lira G., Madrigal-De La Campa A. (2001). Asian-American Variants of Human Papillomavirus 16 and Risk for Cervical Cancer: A Case-Control Study. J. Natl. Cancer Inst..

[91] Alfaro A., Juárez-Torres E., Medina-Martínez I., Mateos-Guerrero N., Bautista-Huerta M., Román-Bassaure E., Villegas-Sepúlveda N., Berumen J. (2016). Different Association of Human Papillomavirus 16 Variants with Early and Late Presentation of Cervical Cancer. PLoS ONE.

[92] González-Losa M.D.R., Mier Y., Teran M.A.L., Puerto-Solís M., García-Carrancá A. (2004). Molecular Variants of HPV Type 16 E6 among Mexican Women with LSIL and Invasive Cancer. J. Clin. Virol. Off. Publ. Pan. Am. Soc. Clin. Virol..

[93] Lizano M., De la Cruz-Hernández E., Carrillo-García A., García-Carrancá A., Ponce de Leon-Rosales S., Dueñas-González A., Hernández-Hernández D.M., Mohar A. (2006). Distribution of HPV16 and 18 Intratypic Variants in Normal Cytology, Intraepithelial Lesions, and Cervical Cancer in a Mexican Population. Gynecol. Oncol..

[94] Escobar-Escamilla N., González-Martínez B.E., Araiza-Rodríguez A., Fragoso-Fonseca D.E., Pedroza-Torres A., Landa-Flores M.G., Garcés-Ayala F., Mendieta-Condado E., Díaz-Quiñonez J.A., Castro-Escarpulli G. (2019). Mutational Landscape and Intra-Host Diversity of Human Papillomavirus Type 16 Long Control Region and E6 Variants in Cervical Samples. Arch. Virol..

[95] Camacho-Ureta E.A., Mendez-Martínez R.S., Vázquez-Vega S., Osuna Martínez U., Sánchez Arenas R., Castillo-Ureta H., Osuna Ramírez I., Torres Montoya E.H., López Moreno H.S., García-Carranca A. (2018). High Frequency of HPV16 European Variant E350G among Mexican Women from Sinaloa. Indian J. Med. Res..

[96] Pérez-Quintanilla M., Méndez-Martínez R., Vázquez-Vega S., Espinosa-Romero R., Sotelo-Regil R., Pérez-Montiel M.D., Ramos-Alamillo U., De Jesús Cabrera-López T., Barquet-Muñoz S.A., Pérez-Plascencia C. (2020). High Prevalence of Human Papillomavirus and European Variants of HPV 16 Infecting Concomitantly to Cervix and Oral Cavity in HIV Positive Women. PLoS ONE.

[97] Calleja-Macias I.E., Kalantari M., Huh J., Ortiz-Lopez R., Rojas-Martinez A., Gonzalez-Guerrero J.F., Williamson A.L., Hagmar B., Wiley D.J., Villarreal L. (2004). Genomic Diversity of Human Papillomavirus-16, 18, 31, and 35 Isolates in a Mexican Population and Relationship to European, African, and Native American Variants. Virology.

[98] Ortiz-Ortiz J., Alarcón-Romero L.D.C., Jiménez-López M.A., Garzón-Barrientos V.H., Calleja-Macías I., Barrera-Saldaña H.A., Leyva-Vázquez M.A., Illades-Aguiar B. (2015). Association of Human Papillomavirus 16 E6 Variants with Cervical Carcinoma and Precursor Lesions in Women from Southern Mexico. Virol. J..

[99] Antaño-Arias R., Del Moral-Hernández O., Ortiz-Ortiz J., Alarcón-Romero L.D.C., Navor-Hernández J.A., Leyva-Vázquez M.A., Jiménez-López M.A., Organista-Nava J., Illades-Aguiar B. (2021). E6/E7 Variants of Human Papillomavirus 16 Associated with Cervical Carcinoma in Women in Southern Mexico. Pathogens.

[100] Artaza-Irigaray C., Flores-Miramontes M.G., Olszewski D., Magaña-Torres M.T., López-Cardona M.G., Leal-Herrera Y.A., Piña-Sánchez P., Jave-Suárez L.F., Aguilar-Lemarroy A. (2017). Genetic Variability in E6, E7 and L1 Genes of Human Papillomavirus 62 and Its Prevalence in Mexico. Infect. Agents Cancer.

[101] Conde-Ferraez L., Pacheco-Arjona R., Canul C.N., Gomez-Carballo J., Ramirez-Prado J.H., Ayora-Talavera G., Del Refugio González-Losa M. (2017). Genetic Variability in E6 and E7 Oncogenes from Human Papillomavirus Type 58 in Mexican Women. Intervirology.

[102] Ortiz-Gutiérrez F., Sánchez-Minutti L., Martínez-Herrera J.F., Torres-Escobar I.D., Pezzat-Said E.B., Márquez-Domínguez L., Grandes-Blanco A.I. (2021). Identification of Genetic Variants of Human Papillomavirus in a Group of Mexican HIV/AIDS Patients and Their Possible Association with Cervical Cancer. Pol. J. Microbiol..

[103] Casas L., Galvan S.C., Ordoñez R.M., Ordoñez O., Lopez N., Guido M., Berumen J. (1999). Asian-American variants of Human Papillomavirus type 16 have extensive mutations in the E2 gene and are highly amplified in cervical carcinomas. Int. J. Cancer.

[104] Ordóñez R.M., Espinosa A.M., Sánchez-González D.J., Armendáriz-Borunda J., Berumen J. (2004). Enhanced Oncogenicity of Asian-American Human Papillomavirus 16 Is Associated with Impaired E2 Repression of E6/E7 Oncogene Transcription. J. Gen. Virol..

[105] Amador-Molina A., González-Montoya J.L., García-Carrancá A., Mohar A., Lizano M. (2013). Intratypic Changes of the E1 Gene and the Long Control Region Affect Ori Function of Human Papillomavirus Type 18 Variants. J. Gen. Virol..

[106] López-Saavedra A., González-Maya L., Ponce-De-León S., García-Carrancá A., Mohar A., Lizano M. (2009). Functional Implication of Sequence Variation in the Long Control Region and E2 Gene among Human Papillomavirus Type 18 Variants. Arch. Virol..

[107] Zacapala-Gómez A.E., Del Moral-Hernández O., Villegas-Sepúlveda N., Hidalgo-Miranda A., Romero-Córdoba S.L., Beltrán-Anaya F.O., Leyva-Vázquez M.A., Alarcón-Romero L.D.C., Illades-Aguiar B. (2016). Changes in Global Gene Expression Profiles Induced by HPV 16 E6 Oncoprotein Variants in Cervical Carcinoma C33-A Cells. Virology.

[108] Fragoso-Ontiveros V., Alvarez-García R.M., Contreras-Paredes A., Vaca-Paniagua F., Herrera L.A., López-Camarillo C., Jacobo-Herrera N., Lizano-Soberón M., Pérez-Plasencia C. (2012). Gene Expression Profiles Induced by E6 from Non-European HPV18 Variants Reveals a Differential Activation on Cellular Processes Driving to Carcinogenesis. Virology.

[109] Perez-Bacho E.G., Beltrán-Anaya F.O., Arechaga-Ocampo E., Hernández-Sotelo D., Garibay-Cerdenares O.L., Illades-Aguiar B., Alarcón-Romero L.D.C., Del Moral-Hernández O. (2022). The E6 Oncoprotein of HPV16 AA-c Variant Regulates Cell Migration through the MINCR/MiR-28-5p/RAP1B Axis. Viruses.

[110] Garibay-Cerdenares O.L., Sánchez-Meza L.V., Encarnación-Guevara S., Hernández-Ortíz M., Martínez-Batallar G., Torres-Rojas F.I., Mendoza-Catalán M.Á., Moral-Hernández O.D., Leyva-Vázquez M.A., Illades-Aguiar B. (2021). Effect of HPV 16 E6 Oncoprotein Variants on the Alterations of the Proteome of C33A Cells. Cancer Genom. Proteom..

[111] Araujo-Arcos L.E., Montaño S., Bello-Rios C., Garibay-Cerdenares O.L., Leyva-Vázquez M.A., Illades-Aguiar B. (2022). Molecular Insights into the Interaction of HPV-16 E6 Variants against MAGI-1 PDZ1 Domain. Sci. Rep..

[112] Vazquez-Vega S., Sanchez-Suarez L.P., Andrade-Cruz R., Castellanos-Juarez E., Contreras-Paredes A., Lizano-Soberon M., Garcia-Carranca A., Benitez Bribiesca L. (2013). Regulation of P14ARF Expression by HPV-18 E6 Variants. J. Med. Virol..

[113] Muñoz-Bello J.O., Olmedo-Nieva L., Castro-Muñoz L.J., Manzo-Merino J., Contreras-Paredes A., González-Espinosa C., López-Saavedra A., Lizano M. (2018). HPV-18 E6 Oncoprotein and Its Spliced Isoform E6*I Regulate the Wnt/β-Catenin Cell Signaling Pathway through the TCF-4 Transcriptional Factor. Int. J. Mol. Sci..

[114] Contreras-Paredes A., De la Cruz-Hernández E., Martínez-Ramírez I., Dueñas-González A., Lizano M. (2009). E6 Variants of Human Papillomavirus 18 Differentially Modulate the Protein Kinase B/Phosphatidylinositol 3-Kinase (Akt/PI3K) Signaling Pathway. Virology.

[115] Olmedo-Nieva L., Muñoz-Bello J.O., Contreras-Paredes A., Lizano M. (2018). The Role of E6 Spliced Isoforms (E6*) in Human Papillomavirus-Induced Carcinogenesis. Viruses.

[116] Bello-Rios C., Montaño S., Garibay-Cerdenares O.L., Araujo-Arcos L.E., Leyva-Vázquez M.A., Illades-Aguiar B. (2021). Modeling and Molecular Dynamics of the 3D Structure of the HPV16 E7 Protein and Its Variants. Int. J. Mol. Sci..

[117] Fuentes-González A.M., Muñoz-Bello J.O., Manzo-Merino J., Contreras-Paredes A., Pedroza-Torres A., Fernández-Retana J., Pérez-Plasencia C., Lizano M. (2019). Intratype Variants of the E2 Protein from Human Papillomavirus Type 18 Induce Different Gene Expression Profiles Associated with Apoptosis and Cell Proliferation. Arch. Virol..

[118] IARC Cancer Today. https://gco.iarc.fr/today/home.

[119] World Health Organization Human Papillomavirus (HPV) Vaccination Coverage. https://immunizationdata.who.int/pages/coverage/hpv.html.

[120] Brotherton J.M.L., Giuliano A.R., Markowitz L.E., Dunne E.F., Ogilvie G.S. (2016). Monitoring the Impact of HPV Vaccine in Males-Considerations and Challenges. Papillomavirus Res..

[121] Maldonado I., Plata M., Gonzalez M., Correa A., Nossa C., Giuliano A.R., Joura E.A., Ferenczy A., Ronnett B.M., Stoler M.H. (2022). Effectiveness, Immunogenicity, and Safety of the Quadrivalent HPV Vaccine in Women and Men Aged 27–45 Years. Hum. Vaccin. Immunother..

[122] Goldstone S.E., Giuliano A.R., Palefsky J.M., Lazcano-Ponce E., Penny M.E., Cabello R.E., Moreira E.D., Baraldi E., Jessen H., Ferenczy A. (2022). Efficacy, Immunogenicity, and Safety of a Quadrivalent HPV Vaccine in Men: Results of an Open-Label, Long-Term Extension of a Randomised, Placebo-Controlled, Phase 3 Trial. Lancet Infect. Dis..

