# Supplementary material for: Epidemiology and Molecular Biology of HPV Variants in Cervical Cancer: The State of the Art in Mexico
Source: Int J Mol Sci. 2022 Aug 2;23(15):8566. doi: 10.3390/ijms23158566 (PMC9368912; doi:10.3390/ijms23158566)
Supplement: Supplementary file 1 [file ijms-23-08566-s001.zip › ijms-1820448-supplementary.pdf]

Supplementary material

1. Nucleotide changes among E6/E7 variants of HR-HPV. Nucleotide sequences were obtained from Papillomavirus Episteme (PaVE) portal [69], then NCBI accession number of each variant was used to analyzed E6/E7 bicistronic sequence and Clustal Omega (v 1.2.4) was used for E6/E7 alignments of **A)** HPV-16; and **B)** HPV-18 variants. E6 and E7 ORF reference genome are depicted in pink and yellow, respectively. Moreover, nucleotide changes of each variant are shown in green; and asterisk (*) represent similitudes in nucleotide sequence among variants while empty spaces represent changes among variants in relation to reference genome. According to sequences obtained from NCBI, broken lines indicate differences in the start coding sites among variants.

A) Nucleotide alignments of E6/E7 HPV-16 variants

Sublineage **Nulceotide sequence E6/E7 HPV-16 bicistron** **Position**

```
A4      ATGCACCAAAAGAGAACTGCAATGTTTCAGGACCCACAGGAGCGACCCAGAAAGTTACCA 60
A3      ---------------------ATGTTTCAGGACCCACAGGAGCGACCCAGAAAGTTACCA 39
A1      ATGCACCAAAAGAGAACTGCAATGTTTCAGGACCCACAGGAGCGACCCAGAAAGTTACCA 60
A2      ATGCACCAAAAGAGAACTGCAATGTTTCAGGACCCACAGGAGCGACCCGGAAAGTTACCA 60
C1      ATGCACCAAAAGAGAACTGCAATGTTCCAGGACCCACAGGAGCGACCCATAAAGTTACCA 60
D2      ATGCACCAAAAGAGAACTGCAATGTTTCAGGACCCACAGGAGCGACCCAGAAAGTTACCA 60
D3      ATGCACCAAAAGAGAACTGCAATGTTTCAGGACCCACAGGAGCGACCCAGAAAGTTACCA 60
D1      ---------------------ATGTTTCAGGACCCACAGGAGCGACCCAGAAAGTTACCA 39
D4      ---------------------ATGTTTCAGGACCCACAGGAGCGACCCAGAAAGTTACCA 39
C2      ---------------------ATGTTTCAGGACCCACAGGAGCGACCCAGAAAGATACCA 39
B4      ---------------------ATGTTTCAGGACCCACAGGAGCGACCCGGAAAGTTACCA 39
B2      ---------------------ATGTTTCAGGACCCACAGGAGCGACCCGGAAAGTTACCA 39
C4      ---------------------ATGTTTCAGGACCCACAGGAGCGACCCAGAAAGTTACCA 39
B3      ---------------------ATGTTTCAGGACCCACAGGAGCGACCCAGAAAGTTACCA 39
B1      CTGCACCAAAAGAGAACTGCAATGTTTCAGGACCCACAGGAGCGACCCACAAAGTTACCA 60
C3      ---------------------ATGTTTCAGGACCCACAGGAGCGACCCAGAAAGTTACCA 39
                             ***** *********************  **** *****
```

Sublineage **Nulceotide sequence E6/E7 HPV-16 bicistron** **Position**

```
A4      CAGTTATGCACAGAGCTGCAAACAACTATACATGAGATAATATTAGAATGTGTGTACTGC 120
A3      CAGTTATGCACAGAGCTGCAAACAACTATACATGATATAATATTAGAATGTGTGTACTGC 99
A1      CAGTTATGCACAGAGCTGCAAACAACTATACATGATATAATATTAGAATGTGTGTACTGC 120
A2      CAGTTATGCACAGAGCTGCAAACAACTATACATGATATAATATTAGAATGTGTGTACTGC 120
C1      GATTTATGCACAGAGCTGCAAACAACTATACATGATATAATATTAGAATGTGTGTACTGC 120
D2      CATTTATGCACAGAGCTGCAAACAACTATACATGATATAATATTAGAATGTGTGTACTGC 120
D3      CATTTATGCACAGAGCTGCAAACAACTATACATGATATAATATTAGAATGTGTGTACTGC 120
D1      CATTTATGCACAGAGCTGCAAACAACTATACATGATATAATATTAGAATGTGTGTACTGC 99
D4      CATTTATGCACAGAGCTGCAAACAACTATACATGATATAATATTAGAATGTGTGTACTGC 99
C2      GATTTATGCACAGAGCTGCAAACAACTATACATGATATAATATTAGAATGTGTGTACTGC 99
B4      GATTTATGCACAGAGCTGCAAACAACTATACATGATATAATATTAGAATGTGTGTACTGC 99
B2      GATTTATGCACAGAGCTGCAAACAACTATACATGATATAATATTAGAATGTGTGTACTGC 99
C4      GATTTATGCACAGAGCTGCAAACAACTATACATGATATAATATTAGAATGTGTGTACTGC 99
B3      GATTTATGCACAGAGCTGCAAACAACTATACATGATATAATATTAGAATGTGTGTACTGC 99
B1      GATTTATGCACAGAGCTGCAAACAACTATACATGATATAATATTAGAATGTGTGTACTGC 120
C3      GATTTATGCACAGAGCTGCAAACAACTATACATGATATAATATTAGAATGTGTGTACTGC 99
         * ******************************** ************************
```

Sublineage **Nulceotide sequence E6/E7 HPV-16 bicistron** **Position**

```
A4      AAGCAACAGTTACTGCGACGTGAGGTATATGACTTTGCTTTTCGGGATTTATGCATAGTA 180
A3      AAGCAACAGTTACTGCGACGTGAGGTATATGACTTTGCTTTTCGGGATTTATGCATAGTA 159
A1      AAGCAACAGTTACTGCGACGTGAGGTATATGACTTTGCTTTTCGGGATTTATGCATAGTA 180
```

```
A2      AAGCAACAGTTACTGCGACGTGAGGTATATGACTTTGCTTTTCGGGATTTATGCATAGTA 180
C1      AAGCAACAGTTACTGCGACGTGAGGTATATGACTTTGCTTTTCGGGATTTATGCATAGTA 180
D2      AAGCAACAGTTACTGCGACGTGAGGTATATGACTTTGCTTTTCGGGATTTATGCATAGTA 180
D3      AAGCAACAGTTACTGCGACGTGAGGTATATGACTTTGCTTTTCGGGATTTATGCATAGTA 180
D1      AAGCAACAGTTACTGCGACGTGAGGTATATGACTTTGCTTTTCGGGATTTATGCATAGTA 159
D4      AAGCAACAGTTACTGCGACGTGAGGTATATGACTTTGCTTTTCGGGATTTATGCATAGTA 159
C2      AAGCAACAGTTACTGCGACGTGAGGTATATGACTTTGCTTTTCGGGATTTATGCATAGTA 159
B4      AAGCAACAGTTACTGCGACGTGAGGTATATGACTTTGCTTTTCGGGATTTATGCATAGTA 159
B2      AAGCAACAGTTACTGCGACGTGAGGTATATGACTTTGCTTTTCGGGATTTATGCATAGTA 159
C4      AAGCAACAGTTACTGCGACGTGAGGTATATGACTTTGCTTTTCGGGATTTATGCATAGTA 159
B3      AAGCAACAGTTACTGCGACGTGAGGTATATGACTTTGCTTTTCGGGATTTATGCATAGTA 159
B1      AAGCAACAGTTACTGCGACGTGAGGTATATGACTTTGCTTTTCGGGATTTATGCATAGTA 180
C3      AAGCAACAGTTACTGCGACGTGAGGTATATGACTTTGCTTTTCGGGATTTATGCATAGTA 159
        ************************************************************
```

Sublineage **Nulceoteide sequence E6/E7 HPV-16 bicistron** **Position**

```
A4      TATAGAGATGGGAATCCATATGCTGTATGTGATAAATGTTTAAAGTTTTATTCTAAAATT 240
A3      TATAGAGATGGGAGTCCATATGCTGTATGTGATAAATGTTTAAAGTTTTATTCTAAAATT 219
A1      TATAGAGATGGGAATCCATATGCTGTATGTGATAAATGTTTAAAGTTTTATTCTAAAATT 240
A2      TATAGAGATGGGAATCCATATGCTGTATGTGATAAATGTTTAAAGTTTTATTCTAAAATT 240
C1      TATAGAGATGGGAATCCATATGCAGTGTGTGATAAATGTTTAAAGTTTTATTCTAAAATT 240
D2      TATAGAGATGGGAATCCATATGCAGTGTGTGATAAATGTTTAAAGTTTTATTCTAAAATT 240
D3      TATAGAGATGGGAATCCATATGCAGTGTGTGATAAATGTTTAAAGTTTTATTCTAAAATT 240
D1      TATAGAGATGGGAATCCATATGCAGTGTGTGATAAATGTTTAAAGTTTTATTCTAAAATT 219
D4      TATAGAGATGGGAATCCATATGCAGTGTGTGATAAATGTTTAAAGTTTTATTCTAAAATT 219
C2      TATAGAGATGGGAATCCATATGGAGTGTGTGATAAATGTTTAAAGTTTTATTCTAAAATT 219
B4      TATAGAGATGGGAATCCATATGCAGTGTGTGAGAAATGTTTAAAGTTTTATTCTAAAATT 219
B2      TATAGAGATGGGAATCCATATGCAGTGTGTGATAAATGTTTAAAGTTTTATTCTAAAATT 219
C4      TATAGAGATGGGAATCCATATGCAGTGTGTGATAAATGTTTAAAGTTTTATTCTAAAATT 219
B3      TATAGAGATGGGAATCCATATGCAGTGTGTGAGAAATGTTTAAAGTTTTATTCTAAAATT 219
B1      TATAGAGATGGGAATCCATATGCAGTGTGTGATAAATGTTTAAAGTTTTATTCTAAAATT 240
C3      TATAGAGATGGGAATCCATATGCAGTGTGTGATAAATGTTTAAAGTTTTATTCTAAAATT 219
        ************* ********  ** ***** ***************************
```

Sublineage **Nulceoteide sequence E6/E7 HPV-16 bicistron** **Position**

```
A4      AGTGAGTATAGACATTATTGTTATAGTTTGTATGGAACAACATTAGAACAGCAATACAAC 300
A3      AGTGAGTATAGACATTATTGTTATAGTTTGTATGGAACAACATTAGAACAGCAATACAAC 279
A1      AGTGAGTATAGACATTATTGTTATAGTTTGTATGGAACAACATTAGAACAGCAATACAAC 300
A2      AGTGAGTATAGACATTATTGTTATAGTGTGTATGGAACAACATTAGAACAGCAATACAAC 300
C1      AGTGAGTATAGATATTATTGTTATAGTTTGTATGGAACAACATTAGAACAGCAATACAAC 300
D2      AGTGAGTATAGATATTATTGTTATAGTGTGTATGGAACAACATTAGAACAGCAATACAAC 300
D3      AGTGAGTATAGATATTATTGTTATAGTGTGTATGGAACAACATTAGAACAGCAATACAAC 300
D1      AGTGAGTATAGATATTATTGTTATAGTGTGTATGGAACAACATTAGAACAGCAATACAAC 279
D4      AGTGAGTATAGATATTATTGTTATAGTGTGTATGGAACAACATTAGAACAGCAATACAAC 279
C2      AGTGAGTATAGATATTATTGTTATAGTTTGTATGGAACAACATTAGAACAGCAATACAAC 279
B4      AGTGAGTATAGATATTATTGTTATAGTGTGTATGGAACAACATTAGAACAGCAATACAAC 279
B2      AGTGAGTATAGATATTATTGTTATAGTTTGTATGGAACAACATTAGAACAGCAATACAAC 279
C4      AGTGAGTATAGATATTATTGTTATAGTTTGTATGGAACAACATTAGAACAGCAATACAAC 279
B3      AGTGAGTATAGATATTATTGTTATAGTTTGTATGGAACAACATTAGAACAGCAATACAAC 279
B1      AGTGAGTATAGATATTATTGTTATAGTTTGTATGGAACAACATTAGAACAGCAATACAAC 300
C3      AGTGAGTATAGATATTATTGTTATAGTTTGTATGGAACAACATTAGAACAGCAATACAAC 279
        ************ ************** ********************************
```

Sublineage **Nulceoteide sequence E6/E7 HPV-16 bicistron** **Position**

```
A4      AAACCGTTGTGTGATTTGTTAATTAGGTGTATTAACTGTCAAAAGCCACTGTGTCCTGAA 360
A3      AAACCGTTGTGTGATTTGTTAATTAGGTGTATTAACTGTCAAAAGCCACTGTGTCCTGAA 339
```

A1	AAACCGTTGTGTGATTTGTTAATTAGGTGTATTAACTGTCAAAAGCCACTGTGTCCTGAA	360
A2	AAACCGTTGTGTGATTTGTTAATTAGGTGTATTAACTGTCAAAAGCCACTGTGTCCTGAA	360
C1	AAACCGTTGTGTGATTTGTTGATTAGGTGTATTAACTGTCAAAAGCCACTGTGTCCTGAA	360
D2	AAACCGTTGTGTGATTTGTTAATTAGGTGTATTAACTGTCAAAAGCCACTGTGTCCTGAA	360
D3	AAACCGTTGTGTGATTTGTTAATTAGGTGTATTAACTGTCAAAAGCCACTATGTCCTGAA	360
D1	AAACCGTTGTGTGATTTGTTAATTAGGTGTATTAACTGTCAAAAGCCACTGTGTCCTGAA	339
D4	AAACCGTTGTGTGATTTGTTAATTAGGTGTATTAACTGTCAAAAGCCACTGTGTCCTGAA	339
C2	AAACCGTTGTGTGATTTGTTAATTAGGTGTATTAACTGTCAAAAGCCACTGTGTCCTGAA	339
B4	AAACCGTTGTGTGATTTGTTAATTAGGTGTATTAACTGTCAAAAGCCACTGTGTCCTGAA	339
B2	AAACCGTTGTGTGATTTGTTAATTAGGTGTATTAACTGTCAAAAGCCACTGTGTCCTGAA	339
C4	AAACCGTTGTGTGATTTGTTAATTAGGTGTATTAACTGTCAAAAGCCACTGTGTCCTGAA	339
B3	AAACCGTTGTGTGATTTGTTAATTAGGTGTATTAACTGTCAAAAGCCACTGTGTCCTGAA	339
B1	AAACCGTTGTGTGATTTGTTAATTAGGTGTATTAACTGTCAAAAGCCACTGTGTCCTGAA	360
C3	AAACCGTTGTGTGATTTGTTAATTAGGTGTATTAACTGTCAAAAGCCACTGTGTCCTGAA	339
	******************** **************************** **********	

Sublineage	Nulceotide sequence E6/E7 HPV-16 bicistron	Position
A4	GAAAAGCAAAGACATCTGGACAAAAAGCAAAGATTCCATAATATAAGGGGTCGGTGGACC	420
A3	GAAAAGCAAAGACATCTGGACAAAAAGCAAAGATTCCATAATATAAGGGGTCGGTGGACC	399
A1	GAAAAGCAAAGACATCTGGACAAAAAGCAAAGATTCCATAATATAAGGGGTCGGTGGACC	420
A2	GAAAAGCAAAGACATCTGGACAAAAAGCAAAGATTCCATAATATAAGGGGTCGGTGGACC	420
C1	GAAAAGCAAAGACATCTGGACAAAAAGCAAAGATTCCATAATATAAGGGGTCGGTGGACC	420
D2	GAAAAGCAAAGACATCTGGACAAAAAGCAAAGATTCCATAATATAAGGGGTCGGTGGACC	420
D3	GAAAAGCAAAGACATCTGGACAAAAAGCAAAGATTCCATAATATAAGGGGTCGGTGGACC	420
D1	GAAAAGCAAAGACATCTGGACAAAAAGCAAAGATTCCATAATATAAGGGGTCGGTGGACC	399
D4	GAAAAGCAAAGACATCTGGACAAAAAGCAAAGATTCCATAATATAAGGGGTCGGTGGACC	399
C2	GAAAAGCAAAGACATCTGGACAAAAAGCAAAGATTCCATAATATAAGGGGTCGGTGGACC	399
B4	GAAAAGCAAAGACATCTGGACAAAAAGCAAAGATTCCATAATATAAGGGGTCGGTGGACC	399
B2	GAAAAGCAAAGACATCTGGACAAAAAGCAAAGATTCCATAATATAAGGGGTCGGTGGACC	399
C4	GAAAAGCAAAGACATCTGGACAAAAAGCAAAGATTCCATAATATAAGGGGTCGGTGGACC	399
B3	GAAAAGCAAAGACATCTGGACAAAAAGCAAAGATTCCATAATATAAGGGGTCGGTGGACC	399
B1	GAAAAGCAAAGACATCTGGACAAAAAGCAAAGATTCCATAATATAAGGGGTCGGTGGACC	420
C3	GAAAAGCAAAGACATCTGGACAAAAAGCAAAGATTCCATAATATAAGGGGTCGGTGGACC	399
	**	

Sublineage	Nulceotide sequence E6/E7 HPV-16 bicistron	Position
A4	GGTCGATGTATGTCTTGTTGCAGATCATCAAGAACACGTAGAGAAACCCAGCTGTAATCA	480
A3	GGTCGATGTATGTCTTGTTGCAGATCATCAAGAACACGTAGAGAAACCCAGCTGTAATCA	459
A1	GGTCGATGTATGTCTTGTTGCAGATCATCAAGAACACGTAGAGAAACCCAGCTGTAATCA	480
A2	GGTCGATGTATGTCTTGTTGCAGATCATCAAGAACACGTAGAGAAACCCAGCTGTAATCA	480
C1	GGTCGATGTATGTCTTGTTGCAGATCATCAAGAACACGTAGAGAAACCCAGCTGTAATCA	480
D2	GGTCGATGTATGTCTTGTTGCAGATCATCGAGAACACGTAGAGAAACCCAGCTGTAATCA	480
D3	GGTCGATGTATGTCTTGTTGCAGATCATCGAGAACACGTAGAGAAACCCAGCTGTAATCA	480
D1	GGTCGATGTATGTCTTGTTGCAGATCATCAAGAACACGTAGAGAAACCCAGCTGTAATCA	459
D4	GGTCGATGTATGTCTTGTTGCAGATCATCAAGAACACGTAGAGAAACCCAGCTGTAATCA	459
C2	GGTCGATGTATGTCTTGTTGCAGATCATCAAGAACACGTAGAGAAACCCAGCTGTAATCA	459
B4	GGTCGATGTATGTCTTGTTGCAGATCATCAAGAACACGTAGAGAAACCCAGCTGTAATCA	459
B2	GGTCGATGTATGTCTTGTTGCAGATCATCAAGAACACGTAGAGAAACCCAGCTGTAATCA	459
C4	GGTCGATGTATGTCTTGTTGCAGATCATCAAGAACACGTAGAGAAACCCAGCTGTAATCA	459
B3	GGTCGATGTATGTCTTGTTGCAGATCATCAAGAACACGTAGAGAAACCCAGCTGTAATCA	459
B1	GGTCGATGTATGTCTTGTTGCAGATCATCAAGAACACGTAGAGAAACCCAGCTGTAATCA	480
C3	GGTCGATGTATGTCTTGTTGCAGATCATCAAGAACACGTAGAGAAACCCAGCTGTAATCA	459
	***************************** ******************************	

Sublineage	Nulceotide sequence E6/E7 HPV-16 bicistron	Position
A4	TGCATGGAGATACACCTACATTGCATGAATATATGTTAGATTTGCAACCAGAGACAACTG	540
A3	TGCATGGAGATACACCTACATTGCATGAATATATGTTAGATTTGCAACCAGAGACAACTG	519
A1	TGCATGGAGATACACCTACATTGCATGAATATATGTTAGATTTGCAACCAGAGACAACTG	540
A2	TGCATGGAGATACACCTACATTGCATGAATATATGTTAGATTTGCAACCAGAGACAACTG	540
C1	TGCATGGAGATACACCTACATTGCATGAATATATGTTAGATTTGCAACCAGAGACAACTG	540
D2	TGCATGGAGATACACCTACATTGCATGAATATATGTTAGATTTGCAACCAGAGACAACTG	540
D3	TGCATGGAGATACACCTACATTGCATGAATATATGTTAGATTTGCAACCAGAGACAACTG	540
D1	TGCATGGAGATACACCTACATTGCATGAATATATGTTAGATTTGCAACCAGAGACAACTG	519
D4	TGCATGGAGATACACCTACATTGCATGAATATATGTTAGATTTGCAACCAGAGACAACTG	519
C2	TGCATGGAGATACACCTACATTGCATGAATATATGTTAGATTTGCAACCAGAGACAACTG	519
B4	TGCATGGAGATACACCTACATTGCATGAATATATGTTAGATTTGCAACCAGAGACAACTG	519
B2	TGCATGGAGATACACCTACATTGCATGAATATATGTTAGATTTGCAACCAGAGACAACTG	519
C4	TGCATGGAGATACACCTACATTGCATGAATATATGTTAGATTTGCAACCAGAGACAACTG	519
B3	TGCATGGAGATACACCTACATTGCATGAATATATGTTAGATTTGCAACCAGAGACAACTG	519
B1	TGCATGGAGATACACCTACATTGCATGAATATATGTTAGATTTGCAACCAGAGACAACTG	540
C3	TGCATGGAGATACACCTACATTGCATGAATATATGTTAGATTTGCAACCAGAGACAACTG	519
	**	

Sublineage	Nulceotide sequence E6/E7 HPV-16 bicistron	Position
A4	ATCTCTACTGTTATGAGCAATTAAGTGACAGCTCAGAGGAGGAGGATGAAATAGATGGTC	600
A3	ATCTCTACTGTTATGAGCAATTAAATGACAGCTCAGAGGAGGAAGATGAAATAGATGGTC	579
A1	ATCTCTACTGTTATGAGCAATTAAATGACAGCTCAGAGGAGGAGGATGAAATAGATGGTC	600
A2	ATCTCTACTGTTATGAGCAATTAAATGACAGCTCAGAGGAGGAGGATGAAATAGATGGTC	600
C1	ATCTCTACTGTTATGAGCAATTAAGTGACAGCTCAGAGGAGGAGGATGAAATAGATGGTC	600
D2	ATCTCTACTGTTATGAGCAATTAAATGACAGCTCAGAGGAGGAGGATGAAATAGATGGTC	600
D3	ATCTCTACTGTTATGAGCAATTAAATGACAGCTCAGAGGAGGAGGATGAAATAGATGGTC	600
D1	ATCTCTACTGTTATGAGCAATTAAATGACAGCTCAGAGGAGGAGGATGAAATAGATGGTC	579
D4	ATCTCTACTGTTATGAGCAATTAAATGACAGCTCAGAGGAGGAGGATGAAATAGATGGTC	579
C2	ATCTCTACTGTTATGAGCAATTAAATGACAGCTCAGAGGAGGAGGATGAAATAGATGGTC	579
B4	ATCTCTACTGTTATGAGCAATTAAATGACAGCTCAGAGGAGGAGGATGAAATAGATGGTC	579
B2	ATCTCTACTGTTATGAGCAATTAAGTGACAGCTCAGAGGAGGAGGATGAAATAGATGGTC	579
C4	ATCTCTACTGTTATGAGCAATTAAATGACAGCTCAGAGGAGGAGGATGAAATAGATGGTC	579
B3	ATCTCTACTGTTATGAGCAATTAAATGACAGCTCAGAGGAGGAGGATGAAATAGATGGTC	579
B1	ATCTCTACTGTTATGAGCAATTAAATGACAGCTCAGAGGAGGAGGATGAAATAGATGGTC	600
C3	ATCTCTACTGTTATGAGCAATTAAATGACAGCTCAGAGGAGGAGGATGAAATAGATGGTC	579
	************************ ****************** ****************	

Sublineage	Nulceotide sequence E6/E7 HPV-16 bicistron	Position
A4	CAGCTGGACAAGCAGAACCGGACAGAGCCCATTACAATATTGTAACCTTTTGTTGCAAGT	660
A3	CAGCTGGACAAGCAGAACCGGACAGAGCCCATTACAATATTGTAACCTTTTGTTGCAAGT	639
A1	CAGCTGGACAAGCAGAACCGGACAGAGCCCATTACAATATTGTAACCTTTTGTTGCAAGT	660
A2	CAGCTGGACAAGCAGAACCGGACAGAGCCCATTACAATATTGTAACCTTTTGTTGCAAGT	660
C1	CAGCTGGACAAGCAGAACCGGACAGAGCCCATTACAATATTGTAACCTTTTGTTGCAAGT	660
D2	CAGCTGGACAAGCAGAACCGGACAGAGCCCATTACAATATTGTAACCTTCTGTTGCAAGT	660
D3	CAGCTGGACAAGCAGAACCGGACAGAGCCCATTACAATATTGTAACCTTCTGTTGCAAGT	660
D1	CAGCTGGACAAGCAGAACCGGACAGAGCCCATTACAATATTGTAACCTTTTGTTGCAAGT	639
D4	CAGCTGGACAAGCAGAACCGGACAGAGCCCATTACAATATTGTAACCTTTTGTTGCAAGT	639
C2	CAGCTGGACAAGCAGAACCGGACAGAGCCCATTACAATATTGTAACCTTTTGTTGCAAGT	639
B4	CAGCTGGACAAGCAGAACCGGACAGAGCCCATTACAATATTGTAACCTTTTGTTGCAAGT	639
B2	CAGCTGGACAAGCAGAACCGGACAGAGCCCATTACAATATTGTAACCTTTTGTTGCAAGT	639
C4	CAGCTGGACAAGCAGAACCGGACAGAGCCCATTACAATATTGTAACCTTTTGTTGCAAGT	639
B3	CAGCTGGACAAGCAGAACCGGACAGAGCCCATTACAATATTGTAACCTTTTGTTGCAAGT	639
B1	CAGCTGGACAAGCAGAACCGGACAGAGCCCATTACAATATTGTAACCTTTTGTTGCAAGT	660

```
C3        CAGCTGGACAAGCAGAACCGGACAGAGCCCATTACAATATTGTAACCTTTTGTTGCAAGT 639
          ************************************************* **********
```

Sublineage	Nulceotide sequence E6/E7 HPV-16 bicistron	Position

```
A4        GTGACTCTACGCTTCGGTTGTGCGTACAAAGCACACACGTAGACATTCGTACTTTGGAAG 720
A3        GTGACTCTACGCTTCGGTTGTGCGTACAAAGCACACACGTAGACATTCGTACTTTGGAAG 699
A1        GTGACTCTACGCTTCGGTTGTGCGTACAAAGCACACACGTAGACATTCGTACTTTGGAAG 720
A2        GTGACTCTACGCTTCGGTTGTGCGTACAAAGCACACACGTAGACATTCGTACTTTGGAAG 720
C1        GTGACTCTACGCTTCGGTTGTGCGTACAAAGCACACACGTAGACATCCGTACGTTGGAAG 720
D2        GTGACTCTACGCTTCGGTTGTGCGTACAAAGCACACACGTAGACATCCGTACGTTGGAAG 720
D3        GTGACTCTACGCTTCGGTTGTGCGTACAAAGCACACACGTAGACATCCGTACGTTGGAAG 720
D1        GTGACTCTACGCTTCGGTTGTGCGTACAAAGCACACACGTAGACATCCGTACGTTGGAAG 699
D4        GTGACTCTACGCTTCGGTTGTGCGTACAAAGCACACACGTAGACATCCGTACGTTGGAAG 699
C2        GTGACTCTACGCTTCGGTTGTGCGTACAAAGCACACACGTAGACATCCGTACGTTGGAAG 699
B4        GTGACTCTACGCTTCGGTTGTGCGTACAAAGCACACACGTAGACATCCGTACGTTGGAAG 699
B2        GTGACTCTACGCTTCGGTTGTGCGTACAAAGCACACACGTAGACATCCGTACGTTGGAAG 699
C4        GTGACTCTACGCTTCGGTTGTGCGTACAAAGCACACACGTAGACATCCGTACGTTGGAAG 699
B3        GTGACTCTACGCTTCGGTTGTGCGTACAAAGCACACACGTAGACATCCGTACGTTGGAAG 699
B1        GTGACTCTACGCTTCGGTTGTGCGTACAAAGCACACACGTAGACATCCGTACGTTGGAAG 720
C3        GTGACTCTACGCTTCGGTTGTGCGTACAAAGCACACACGTAGACATCCGTACGTTGGAAG 699
          ********************************************** ***** *******
```

Sublineage	Nulceotide sequence E6/E7 HPV-16 bicistron	Position

```
A4        ACCTGTTAATGGCACACTAGGAATTGTGTGCCCCATCTGTTCCCAGAAACCATAA      776
A3        ACCTGTTAATGGCACACTAGGAATTGTGTGCCCCATCTGTTCTCAGAAACCATAA      755
A1        ACCTGTTAATGGCACACTAGGAATTGTGTGCCCCATCTGTTCTCAGAAACCATAA      776
A2        ACCTGTTAATGGCACACTAGGAATTGTGTGCCCCATCTGTTCTCAGAAACCATAA      776
C1        ACCTGTTAATGGCACACTAGGAATTGTGTGCCCCATCTGTTCTCAGAAACCATAA      776
D2        ACCTGTTAATGGCACACTAGGAATTGTGTGCCCCATCTGTTCTCAGAAACCATAA      776
D3        ACCTGTTAATGGCACACTAGGAATTGTGTGCCCCATCTGTTCTCAGAAACCATAA      776
D1        ACCTGTTAATGGCACACTAGGAATTGTGTGCCCCATCTGTTCTCAGAAACCATAA      755
D4        ACCTGTTAATGGCACACTAGGAATTGTGTGCCCCATCTGTTCTCAGAAACCATAA      755
C2        ACCTGTTAATGGCACACTAGGAATTGTGTGCCCCATCTGTTCTCAGAAACCATAA      755
B4        ACCTGTTAATGGCACACTAGGAATTGTGTGCCCCATCTGTTCTCAGAAACCATAA      755
B2        ACCTGTTAATGGCACACTAGGAATTGTGTGCCCCATCTGTTCTCAGAAACCATAA      755
C4        ACCTGTTAATGGCACACTAAGAATTGTGTGCCCCATCTGTTCTCAGAAACCATAA      755
B3        ACCTGTTAATGGCACACTAGGAATTGTGTGCCCCATCTGTTCTCAGAAACCATAA      755
B1        ACCTGTTAATGGCACACTAGGAATTGTGTGCCCCATCTGTTCTCAGAAACCATAA      776
C3        ACCTGTTAATGGCACACTAGGAATTGTGTGCCCCATCTGTTCTCAGAAACCATAA      755
          ******************* ********************** ************
```

B) Nucleotide alignments of E6/E7 HPV-18 variants

Sublineage	Nulceotide sequence E6/E7 HPV-18 bicistron	Position

```
A3        ATGGCGCGCTTTGAGGATCCAACACGGCGACCCTACAAGCTACCTGATCTGTGCACGGAA 60
A5        ATGGCGCGCTTTGAGGATCCAACACGGCGACCCTACAAGCTACCCGATCTGTGCACGGAA 60
A4        ATGGCGCGCTTTGAGGATCCAACACGGCGACCCTACAAGCTACCTGATCTGTGCACGGAA 60
A1        ATGGCGCGCTTTGAGGATCCAACACGGCGACCCTACAAGCTACCTGATCTGTGCACGGAA 60
A2        ATGGCGCGCTTTGAGGATCCAACACGGCGACCCTACAAGCTACCTGATCTGTGCACGGAA 60
B1        ATGGCGCGCTTTGAGGATCCAACACGGCGACCCTACAAGCTACCTGATCTGTGCACGGAA 60
C1        ATGGCGCGCTTTGAGGATCCAACACGGCGACCCTACAAGCTACCTGATCTGTGCACGGAA 60
B2        ATGGCGCGCTTTGAGGATCCAACACGGCGACCCTACAAGCTACCTGATCTGTGCACGGAA 60
```

```
B3        ATGGCGCGCTTTGAGGATCCAACACGGCGACCCTACAAGCTACCTGATCTGTGCACGGAA 60
          ******************************************** ***************
```

Sublineage **Nulceotide sequence E6/E7 HPV-18 bicistron** **Position**

```
A3        CTGAACACTTCACTGCAAGACATAGAAATAACCTGTGTATATTGCAAGACAGTATTGGAA 120
A5        CTGAACACTTCACTGCAAGACATAGAAATAACCTGTGTATATTGCAAGACAGTATTGGAA 120
A4        CTGAACACTTCACTGCAAGACATAGAAATAACCTGTGTATATTGCAAGACAGTATTGGAA 120
A1        CTGAACACTTCACTGCAAGACATAGAAATAACCTGTGTATATTGCAAGACAGTATTGGAA 120
A2        CTGAACACTTCACTGCAAGACATAGAAATAACCTGTGTATATTGCAAGACAGTATTGGAA 120
B1        CTGAACACTTCACTGCAAGACATAGAAATAACCTGTGTATATTGCAAGACAGTATTGGAA 120
C1        CTGAACACTTCACTGCAAGACATAGAAATAACCTGTGTATATTGCAAGACAGTATTGGAA 120
B2        CTGAACACTTCACTGCAAGACATAGAAATAACCTGTGTATATTGCAAGACAGTATTGGAA 120
B3        CTGAACACTTCACTGCAAGACATAGAAATAACCTGTGTATATTGCAAGACAGTATTGGAA 120
          ************************************************************
```

Sublineage **Nulceotide sequence E6/E7 HPV-18 bicistron** **Position**

```
A3        CTTACAGGGGTATTTGAATTTGCATTTAAAGATTTATTTGTGGTGTATAGAGACAGTATA 180
A5        CTTACAGAGGTATTTGAATTTGCATTTAAAGATTTATTTGTGGTGTATAGAGACAGTATA 180
A4        CTTACAGAGGTATTTGAATTTGCATTTAAAGATTTATTTGTGGTGTATAGAGACAGTATA 180
A1        CTTACAGAGGTATTTGAATTTGCATTTAAAGATTTATTTGTGGTGTATAGAGACAGTATA 180
A2        CTTACAGAGGTATTTGAATTTGCATTTAAAGATTTATTTGTGGTGTATAGAGACAGTATA 180
B1        CTTACAGAGGTATTTGAATTTGCATTCAAAGATTTATTTGTAGTGTATAGAGACAGTATA 180
C1        CTTACAGAGGTATTTGAATTTGCATTCAAAGATTTATTTGTAGTGTATAGAGACAGTATA 180
B2        CTTACAGAGGTATTTGAATTTGCATTCAAAGATTTATTTGTAGTGTATAGAGACAGTATA 180
B3        CTTACAGAGGTATTTGAATTTGCATTCAAAGATTTATTTGTAGTGTATAGAGACAGTATA 180
          ******* ****************** ************** ******************
```

Sublineage **Nulceotide sequence E6/E7 HPV-18 bicistron** **Position**

```
A3        CCGCATGCTGCATGCCATAAATGTATAGATTTTTATTCTAGAATTAGAGAATTAAGACAT 240
A5        CCGCATGCTGCATGCCATAAATGTATAGATTTTTATTCTAGAATTAGAGAATTAAGACAT 240
A4        CCGCATGCTGCATGCCATAAATGTATAGATTTTTATTCTAGAATTAGAGAATTAAGACAT 240
A1        CCGCATGCTGCATGCCATAAATGTATAGATTTTTATTCTAGAATTAGAGAATTAAGACAT 240
A2        CCGCATGCTGCATGCCATAAATGTATAGATTTTTATTCTAGAATTAGAGAATTAAGACAT 240
B1        CCGCATGCTGCATGCCATAAATGTATAGATTTCTATTCTAGAATTAGAGAATTAAGATAT 240
C1        CCGCATGCTGCATGCCATAAATGTATAGATTTTTATTCTAGAATTAGAGAATTAAGACAT 240
B2        CCGCATGCTGCATGCCATAAATGTATAGATTTTTATTCTAGAATTAGAGAATTAAGACAT 240
B3        CCGCATGCTGCATGCCATAAATGTATAGATTTTTATTCTAGAATTAGAGAATTAAGACAT 240
          ******************************** ************************ **
```

Sublineage **Nulceotide sequence E6/E7 HPV-18 bicistron** **Position**

```
A3        TATTCAGACTCTGTGTATGGAGACACATTGGAAAAACTAACTAACACTGGGTTATACAAT 300
A5        TATTCAGACTCTGTGTATGGAGACACATTGGAGAAACTAACTAACACTGGGTTATACAAT 300
A4        TATTCAGACTCTGTGTATGGAGACACATTGGAAAAACTAACTAACACTGGGTTATACAAT 300
A1        TATTCAGACTCTGTGTATGGAGACACATTGGAAAAACTAACTAACACTGGGTTATACAAT 300
A2        TATTCAGACTCTGTGTATGGAGACACATTGGAAAAACTAACTAACACTGGGTTATACAAT 300
B1        TATTCAGACTCTGTGTATGGAGACACATTAGAAAAACTAACTAACACTGGGTTATACAAT 300
C1        TATTCAGACTCTGTGTATGGAGACACATTAGAAAAACTAACTAACACTGGGTTATACAAT 300
B2        TATTCAGACTCTGTGTATGGAGACACATTAGAAAAACTAACTAACACTGGGTTATACAAT 300
B3        TATTCAGACTCTGTGTATGGAGACACATTAGAAAAACTAACTAACACTGGGTTATACAAT 300
          ***************************** ** ***************************
```

Sublineage **Nulceotide sequence E6/E7 HPV-18 bicistron** **Position**

```
A3        TTATTAATAAGGTGCCTGCGGTGCCAGAAACCGTTGAATCCAGCAGAAAAACTTAGACAC 360
A5        TTATTAATAAGGTGCCTGCGGTGCCAGAAACCGTTGAATCCAGCAGAAAAACTTAGACAC 360
A4        TTATTAATAAGGTGCCTGCGGTGCCAGAAACCGTTGAATCCAGCAGAAAAACTTAGACAC 360
```

Sublineage **Nulceotide sequence E6/E7 HPV-18 bicistron** **Position**

```
A1        TTATTAATAAGGTGCCTGCGGTGCCAGAAACCGTTGAATCCAGCAGAAAAACTTAGACAC 360
A2        TTATTAATAAGGTGCCTGCGGTGCCAGAAACCGTTGAATCCAGCAGAAAAACTTAGACAC 360
B1        TTATTAATAAGGTGCCTGCGGTGCCAGAAACCGTTGAATCCAGCAGAAAAACTTAGACAC 360
C1        TTATTAATAAGGTGCCTGCGGTGCCAGAAACCATTGAATCCAGCAGAAAAACTTAGACAC 360
B2        TTATTAATAAGGTGCCTGCGGTGCCAGAAACCGTTGAATCCAGCAGAAAAACTTAGACAC 360
B3        TTATTAATAAGGTGCCTGCGGTGCCAGAAACCGTTGAATCCAGCAGAAAAACTTAGACAC 360
          ******************************** ***************************
```

Sublineage **Nulceotide sequence E6/E7 HPV-18 bicistron** **Position**

```
A3        CTTAATGAAAAACGACGATTCCACAACATAGCTGGGCACTATAGAGGCCAGTGCCATTCG 420
A5        CTTAATGAAAAACGACGATTCCACAACATAGCTGGGCACTATAGAGGCCAGTGCCATTCG 420
A4        CTTAATGAAAAACGACGATTCCACAACATAGCTGGGCACTATAGAGGCCAGTGCCATTCG 420
A1        CTTAATGAAAAACGACGATTTCACAACATAGCTGGGCACTATAGAGGCCAGTGCCATTCG 420
A2        CTTAATGAAAAACGACGATTCCACAACATAGCTGGGCACTATAGAGGCCAGTGCCATTCG 420
B1        CTTAATGAAAAACGACGATTCCACAAAATAGCTGGGCACTATAGAGGCCAGTGCCATTCG 420
C1        CTTAATGAAAAACGACGATTCCACAACATAGCTGGGCACTATAGAGGCCAGTGCCATTCG 420
B2        CTTAATGAAAAACGACGATTCCACAAAATAGCTGGGCACTATAGAGGCCAGTGCCATTCG 420
B3        CTTAATGAAAAACGACGATTCCACAAAATAGCTGGGCACTATAGAGGCCAGTGCCATTCG 420
          ******************** ***** *********************************
```

Sublineage **Nulceotide sequence E6/E7 HPV-18 bicistron** **Position**

```
A3        TGCTGCAACCGAGCACGACAGGAAAGACTCCAACGACGCAGAGAAACACAAGTATAATAT 480
A5        TGCTGCAACCGAGCACGACAGGAAAGACTCCAACGACGCAGAGAAACACAAGTATAATAT 480
A4        TGCTGCAACCGAGCACGACAGGAAAGACTCCAACGACGCAGAGAAACACAAGTATAATAT 480
A1        TGCTGCAACCGAGCACGACAGGAACGACTCCAACGACGCAGAGAAACACAAGTATAATAT 480
A2        TGCTGCAACCGAGCACGACAGGAAAGACTCCAACGACGCAGAGAAACACAAGTATAATAT 480
B1        TGCTGCAACCGAGCACGACAGGAGAGGCTCCAACGACGCAGAGAAACACAAGTATAATAT 480
C1        TGCTGCAACCGAGCACGACAGGAGAGACTCCAACGACGCAGAGAAACACAAGTATAATAT 480
B2        TGCTGCAACCGAGCACGACAGGAGAGACTCCAACGACGCAGAGAAACACAAGTATAATAT 480
B3        TGCTGCAACCGAGCACGACAGGAGAGACTCCAACGACGCAGAGAAACACAAGTATAATAT 480
          ***********************  * *********************************
```

Sublineage **Nulceotide sequence E6/E7 HPV-18 bicistron** **Position**

```
A3        TAAGTATGCATGGACCTAAGGCAACATTGCAAGACATTGTATTGCATTTAGAGCCCCAAA 540
A5        TAAGTATGCATGGACCTAAGGCAACATTGCAAGACATTGTATTGCATTTAGAGCCCCAAA 540
A4        TAAGTATGCATGGACCTAAGGCAACATTGCAAGACATTGTATTGCATTTAGAGCCCCAAA 540
A1        TAAGTATGCATGGACCTAAGGCAACATTGCAAGACATTGTATTGCATTTAGAGCCCCAAA 540
A2        TAAGTATGCATGGACCTAAGGCAACATTGCAAGACATTGTATTGCATTTAGAGCCCCAAA 540
B1        TAAGTATGTATGGACCTAAGGCAACATTGCAAGACATTGTATTGCATTTAGAGCCTCAAA 540
C1        TAAGTATGTATGGACCTAAGGCAACATTGCAAGACATTGTATTGCATTTAGAGCCCCAAA 540
B2        TAAGTATGTATGGACCTAAGGCAACATTGCAAGACATTGTATTGCATTTAGAGCCTCAAA 540
B3        TAAGTATGTATGGACCTAAGGCAACATTGCAAGACATTGTATTGCATTTAGAGCCTCAAA 540
          ******** ********************************************** ****
```

Sublineage **Nulceotide sequence E6/E7 HPV-18 bicistron** **Position**

```
A3        ATGAAATTCCGGTTGACCTTCTATGTCACGAGCAATTAAGCGACTCAGAGGAAGAAAACG 600
A5        ATGAAATTCCGGTTGACCTTCTATGTCACGAGCAATTAAGCGACTCAGAGGAAGAAAACG 600
A4        ATGAAATTCCGGTTGACCTCCTATGTCACGAGCAATTAAGCGACTCAGAGGAAGAAAACG 600
A1        ATGAAATTCCGGTTGACCTTCTATGTCACGAGCAATTAAGCGACTCAGAGGAAGAAAACG 600
A2        ATGAAATTCCGGTTGACCTTCTATGTCACGAGCAATTAAGCGACTCAGAGGAAGAAAACG 600
B1        ATGAAATTCCGGTTGACCTTCTATGTCACGAGCAATTAAGCGACTCAGAGGAAGAAAACG 600
C1        ATGAAATTCCGGTTGACCTTCTATGTCACGAGCAATTAAGCGACTCAGAGGAAGAAAACG 600
B2        ATGACATTCCGGTTGACCTTCTATGTCACGAGCAATTAAGCGACTCAGAGGAAGAAAACG 600
```

```
B3        ATGAAATTCCGGTTGACCTTCTATGTCACGAGCAATTAAGCGACTCAGAGGAAGAAAACG 600
          **** ************** ****************************************
```

Sublineage **Nulceotide sequence E6/E7 HPV-18 bicistron** **Position**

```
A3        ATGAAATAGATGGAGTTAATCATCAACATTTACCAGCCCGACGAGCTGAACCACAACGTC 660
A5        ATGAAATAGATGGAGTTAATCATCAACATTTACCAGCCCGACGAGCCGAACCACAACGTC 660
A4        ATGAAATAGATGGAGTTAATCATCAACATTTACCAGCCCGACGAGCTGAACCACAACGTC 660
A1        ATGAAATAGATGGAGTTAATCATCAACATTTACCAGCCCGACGAGCCGAACCACAACGTC 660
A2        ATGAAATAGATGGAGTTAATCATCAACATTTACCAGCCCGACGAGCCGAACCACAACGTC 660
B1        ATGAAATAGATGGAGTTAATCATCAACATTTGCCAGCCCGACGAGCCGAACCACAACGTC 660
C1        ATGAAATAGATGGAGTTAATCATCAACATTTACCAGCCCGACGAGCCGAACCACAACGTC 660
B2        ATGAAATAGATGGAGTTAATCATCAACATTTACCAGCCCGACGAGCCGAACCACAACGTC 660
B3        ATGAAATAGATGGAGTTAATCATCAACATTTACCAGCCCGACGAGCCGAACCACAACGTC 660
          ******************************* ************** *************
```

Sublineage **Nulceotide sequence E6/E7 HPV-18 bicistron** **Position**

```
A3        ACACAATGTTGTGTATGTGTTGTAAGTGTGAAGCCAGAATTGAGCTAGTAGTAGAAAGCT 720
A5        ACACAATGTTGTGTATGTGTTGTAAGTGTGAAGCCAGAATTGAGCTAGTAGTAGAAAGCT 720
A4        ACACAATGTTGTGTATGTGTTGTAAGTGTGAAGCCAGAATTGAGCTAGTAGTAGAAAGCT 720
A1        ACACAATGTTGTGTATGTGTTGTAAGTGTGAAGCCAGAATTGAGCTAGTAGTAGAAAGCT 720
A2        ACACAATGTTGTGTATGTGTTGTAAGTGTGAAGCCAGAATTGAGCTAGTAGTAGAAAGCT 720
B1        ACACAATGTTGTGTATGTGTTGTAAGTGTGAAGCCAGAATTGAGCTAGTAGTAGAAAGCT 720
C1        ACACAATGTTGTGTATGTGTTGTAAGTGTGAAGCCAGAATTGAGCTAGTAGTAGAAAGCT 720
B2        ACACAATGTTGTGTATGTGTTGTAAGTGTGAAGCCAGAATTGAGCTAGTAGTAGAAAGCT 720
B3        ACACAATGTTGTGTATGTGTTGTAAGTGTGAAGCCAGAATTGAGCTAGTAGTAGAAAGCT 720
          ************************************************************
```

Sublineage **Nulceotide sequence E6/E7 HPV-18 bicistron** **Position**

```
A3        CAGCAGACGACCTTCGAGCATTCCAGCAGCTGTTTCTGAACACCCTGTCTTTTGTGTGTC 780
A5        CAGCAGACGACCTTCGAGCATTCCAGCAGCTGTTTCTGAACACCCTGTCCTTTGTGTGTC 780
A4        CAGCAGACGACCTTCGAGCATTCCAGCAGCTGTTTCTGAACACCCTGTCCTTTGTGTGTC 780
A1        CAGCAGACGACCTTCGAGCATTCCAGCAGCTGTTTCTGAACACCCTGTCCTTTGTGTGTC 780
A2        CAGCAGACGACCTTCGAGCATTCCAGCAGCTGTTTCTGAACACCCTGTCCTTTGTGTGTC 780
B1        CAGCAGACGACCTTCGAGCATTCCAGCAGCTGTTTCTGAGCACCCTGTCCTTTGTGTGTC 780
C1        CAGCAGACGACCTTCGAGCATTCCAGCAGCTGTTTCTGAACACCCTGTCCTTTGTGTGTC 780
B2        CAGCAGACGACCTTCGAGCATTCCAGCAGCTGTTTCTGAGCACCCTGTCCTTTGTGTGTC 780
B3        CAGCAGACGACCTTCGAGCATTCCAGCAGCTGTTTCTGAAAACCCTGTCCTTTGTGTGTC 780
          ***************************************  ******** **********
```

Sublineage **Nulceotide sequence E6/E7 HPV-18 bicistron** **Position**

```
A3        CGTGGTGTGCATCCCAGCAGTAA                                      803
A5        CGTGGTGTGCATCCCAGCAGTAA                                      803
A4        CGTGGTGTGCATCCCAGCAGTAA                                      803
A1        CGTGGTGTGCATCCCAGCAGTAA                                      803
A2        CGTGGTGTGCATCCCAGCAGTAA                                      803
B1        CGTGGTGTGCATCCCAGCAGTAA                                      803
C1        CGTGGTGTGCATCCCAGCAGTAA                                      803
B2        CGTGGTGTGCATCCCAGCAGTAA                                      803
B3        CGTGGTGTGCATCCCAGCAGTAA                                      803
          ***********************
```

2. Amino acid changes among E6 and E7 variants of HR-HPV. HPV variants Sublineage were obtained from Papillomavirus Episteme (PaVE) portal [69], then NCBI accession number of each variant was used to analyzed both E6 and E7 proteins and then alignments were performed using Clustal Omega (v 1.2.4), **A)** Variants of **A)** E6, **B)** E7 of HPV-16; and variants of **C)** E6 and **D)** E7 of HPV-18 are shown. As depicted, E6 reference proteins are illustrated in pink, while in yellow E7 reference protein is shown. Moreover, amino acids changes of each variant are shown in green; and asterisk (*) represent similitudes in protein sequence among variants while empty spaces represent changes among variants in relation to reference protein. According to sequences obtained from NCBI, broken lines indicate differences in the start of translation among variants.

A) Amino acid alignments of E6 HPV-16 variants

```
Sublineage   Protein sequence of E6 HPV-16                                 Position

A2           MHQKRTAMFQDPQERPGKLPQLCTELQTTIHDIILECVYCKQQLLRREVYDFAFRDLCIV 60
A3           -------MFQDPQERPRKLPQLCTELQTTIHDIILECVYCKQQLLRREVYDFAFRDLCIV 53
A1           MHQKRTAMFQDPQERPRKLPQLCTELQTTIHDIILECVYCKQQLLRREVYDFAFRDLCIV 60
A4           MHQKRTAMFQDPQERPRKLPQLCTELQTTIHEIILECVYCKQQLLRREVYDFAFRDLCIV 60
D1           -------MFQDPQERPRKLPHLCTELQTTIHDIILECVYCKQQLLRREVYDFAFRDLCIV 53
D2           MHQKRTAMFQDPQERPRKLPHLCTELQTTIHDIILECVYCKQQLLRREVYDFAFRDLCIV 60
D3           MHQKRTAMFQDPQERPRKLPHLCTELQTTIHDIILECVYCKQQLLRREVYDFAFRDLCIV 60
D4           -------MFQDPQERPRKLPHLCTELQTTIHDIILECVYCKQQLLRREVYDFAFRDLCIV 53
C2           -------MFQDPQERPRKIPDLCTELQTTIHDIILECVYCKQQLLRREVYDFAFRDLCIV 53
B4           -------MFQDPQERPGKLPDLCTELQTTIHDIILECVYCKQQLLRREVYDFAFRDLCIV 53
B3           -------MFQDPQERPRKLPDLCTELQTTIHDIILECVYCKQQLLRREVYDFAFRDLCIV 53
B1           MHQKRTAMFQDPQERPTKLPDLCTELQTTIHDIILECVYCKQQLLRREVYDFAFRDLCIV 60
C1           MHQKRTAMFQDPQERPIKLPDLCTELQTTIHDIILECVYCKQQLLRREVYDFAFRDLCIV 60
B2           -------MFQDPQERPGKLPDLCTELQTTIHDIILECVYCKQQLLRREVYDFAFRDLCIV 53
C3           -------MFQDPQERPRKLPDLCTELQTTIHDIILECVYCKQQLLRREVYDFAFRDLCIV 53
C4           -------MFQDPQERPRKLPDLCTELQTTIHDIILECVYCKQQLLRREVYDFAFRDLCIV 53
                    ********* *:*.**********:****************************

Sublineage   Protein sequence of E6 HPV-16                                 Position

A2           YRDGNPYAVCDKCLKFYSKISEYRHYCYSVYGTTLEQQYNKPLCDLLIRCINCQKPLCPE 120
A3           YRDGSPYAVCDKCLKFYSKISEYRHYCYSLYGTTLEQQYNKPLCDLLIRCINCQKPLCPE 113
A1           YRDGNPYAVCDKCLKFYSKISEYRHYCYSLYGTTLEQQYNKPLCDLLIRCINCQKPLCPE 120
A4           YRDGNPYAVCDKCLKFYSKISEYRHYCYSLYGTTLEQQYNKPLCDLLIRCINCQKPLCPE 120
D1           YRDGNPYAVCDKCLKFYSKISEYRYYCYSVYGTTLEQQYNKPLCDLLIRCINCQKPLCPE 113
D2           YRDGNPYAVCDKCLKFYSKISEYRYYCYSVYGTTLEQQYNKPLCDLLIRCINCQKPLCPE 120
D3           YRDGNPYAVCDKCLKFYSKISEYRYYCYSVYGTTLEQQYNKPLCDLLIRCINCQKPLCPE 120
D4           YRDGNPYAVCDKCLKFYSKISEYRYYCYSVYGTTLEQQYNKPLCDLLIRCINCQKPLCPE 113
C2           YRDGNPYGVCDKCLKFYSKISEYRYYCYSLYGTTLEQQYNKPLCDLLIRCINCQKPLCPE 113
B4           YRDGNPYAVCEKCLKFYSKISEYRYYCYSVYGTTLEQQYNKPLCDLLIRCINCQKPLCPE 113
B3           YRDGNPYAVCEKCLKFYSKISEYRYYCYSLYGTTLEQQYNKPLCDLLIRCINCQKPLCPE 113
B1           YRDGNPYAVCDKCLKFYSKISEYRYYCYSLYGTTLEQQYNKPLCDLLIRCINCQKPLCPE 120
C1           YRDGNPYAVCDKCLKFYSKISEYRYYCYSLYGTTLEQQYNKPLCDLLIRCINCQKPLCPE 120
B2           YRDGNPYAVCDKCLKFYSKISEYRYYCYSLYGTTLEQQYNKPLCDLLIRCINCQKPLCPE 113
C3           YRDGNPYAVCDKCLKFYSKISEYRYYCYSLYGTTLEQQYNKPLCDLLIRCINCQKPLCPE 113
C4           YRDGNPYAVCDKCLKFYSKISEYRYYCYSLYGTTLEQQYNKPLCDLLIRCINCQKPLCPE 113
             ****.**.**:*************:****:******************************

Sublineage   Protein sequence of E6 HPV-16                                 Position

A2           EKQRHLDKKQRFHNIRGRWTGRCMSCCRSSRTRRETQL                        158
A3           EKQRHLDKKQRFHNIRGRWTGRCMSCCRSSRTRRETQL                        151
A1           EKQRHLDKKQRFHNIRGRWTGRCMSCCRSSRTRRETQL                        158
```

```
A4        EKQRHLDKKQRFHNIRGRWTGRCMSCCRSSRTRRETQL      158
D1        EKQRHLDKKQRFHNIRGRWTGRCMSCCRSSRTRRETQL      151
D2        EKQRHLDKKQRFHNIRGRWTGRCMSCCRSSRTRRETQL      158
D3        EKQRHLDKKQRFHNIRGRWTGRCMSCCRSSRTRRETQL      158
D4        EKQRHLDKKQRFHNIRGRWTGRCMSCCRSSRTRRETQL      151
C2        EKQRHLDKKQRFHNIRGRWTGRCMSCCRSSRTRRETQL      151
B4        EKQRHLDKKQRFHNIRGRWTGRCMSCCRSSRTRRETQL      151
B3        EKQRHLDKKQRFHNIRGRWTGRCMSCCRSSRTRRETQL      151
B1        EKQRHLDKKQRFHNIRGRWTGRCMSCCRSSRTRRETQL      158
C1        EKQRHLDKKQRFHNIRGRWTGRCMSCCRSSRTRRETQL      158
B2        EKQRHLDKKQRFHNIRGRWTGRCMSCCRSSRTRRETQL      151
C3        EKQRHLDKKQRFHNIRGRWTGRCMSCCRSSRTRRETQL      151
C4        EKQRHLDKKQRFHNIRGRWTGRCMSCCRSSRTRRETQL      151
          **************************************
```

B) Amino acid alignments of E7 HPV-16 variants

Sublineage | **Protein sequence of E7 HPV-16** | **Position**

```
C4        MHGDTPTLHEYMLDLQPETTDLYCYEQLNDSSEEEDEIDGPAGQAEPDRAHYNIVTFCCK 60
A1        MHGDTPTLHEYMLDLQPETTDLYCYEQLNDSSEEEDEIDGPAGQAEPDRAHYNIVTFCCK 60
A2        MHGDTPTLHEYMLDLQPETTDLYCYEQLNDSSEEEDEIDGPAGQAEPDRAHYNIVTFCCK 60
A3        MHGDTPTLHEYMLDLQPETTDLYCYEQLNDSSEEEDEIDGPAGQAEPDRAHYNIVTFCCK 60
B1        MHGDTPTLHEYMLDLQPETTDLYCYEQLNDSSEEEDEIDGPAGQAEPDRAHYNIVTFCCK 60
B3        MHGDTPTLHEYMLDLQPETTDLYCYEQLNDSSEEEDEIDGPAGQAEPDRAHYNIVTFCCK 60
B4        MHGDTPTLHEYMLDLQPETTDLYCYEQLNDSSEEEDEIDGPAGQAEPDRAHYNIVTFCCK 60
C2        MHGDTPTLHEYMLDLQPETTDLYCYEQLNDSSEEEDEIDGPAGQAEPDRAHYNIVTFCCK 60
C3        MHGDTPTLHEYMLDLQPETTDLYCYEQLNDSSEEEDEIDGPAGQAEPDRAHYNIVTFCCK 60
D1        MHGDTPTLHEYMLDLQPETTDLYCYEQLNDSSEEEDEIDGPAGQAEPDRAHYNIVTFCCK 60
D2        MHGDTPTLHEYMLDLQPETTDLYCYEQLNDSSEEEDEIDGPAGQAEPDRAHYNIVTFCCK 60
D3        MHGDTPTLHEYMLDLQPETTDLYCYEQLNDSSEEEDEIDGPAGQAEPDRAHYNIVTFCCK 60
D4        MHGDTPTLHEYMLDLQPETTDLYCYEQLNDSSEEEDEIDGPAGQAEPDRAHYNIVTFCCK 60
A4        MHGDTPTLHEYMLDLQPETTDLYCYEQLSDSSEEEDEIDGPAGQAEPDRAHYNIVTFCCK 60
B2        MHGDTPTLHEYMLDLQPETTDLYCYEQLSDSSEEEDEIDGPAGQAEPDRAHYNIVTFCCK 60
C1        MHGDTPTLHEYMLDLQPETTDLYCYEQLSDSSEEEDEIDGPAGQAEPDRAHYNIVTFCCK 60
          ****************************.*******************************
```

Sublineage | **Protein sequence of E7 HPV-16** | **Position**

```
C4        CDSTLRLCVQSTHVDIRTLEDLLMGTLRIVCPICSQKP       98
A1        CDSTLRLCVQSTHVDIRTLEDLLMGTLGIVCPICSQKP       98
A2        CDSTLRLCVQSTHVDIRTLEDLLMGTLGIVCPICSQKP       98
A3        CDSTLRLCVQSTHVDIRTLEDLLMGTLGIVCPICSQKP       98
B1        CDSTLRLCVQSTHVDIRTLEDLLMGTLGIVCPICSQKP       98
B3        CDSTLRLCVQSTHVDIRTLEDLLMGTLGIVCPICSQKP       98
B4        CDSTLRLCVQSTHVDIRTLEDLLMGTLGIVCPICSQKP       98
C2        CDSTLRLCVQSTHVDIRTLEDLLMGTLGIVCPICSQKP       98
C3        CDSTLRLCVQSTHVDIRTLEDLLMGTLGIVCPICSQKP       98
D1        CDSTLRLCVQSTHVDIRTLEDLLMGTLGIVCPICSQKP       98
D2        CDSTLRLCVQSTHVDIRTLEDLLMGTLGIVCPICSQKP       98
D3        CDSTLRLCVQSTHVDIRTLEDLLMGTLGIVCPICSQKP       98
D4        CDSTLRLCVQSTHVDIRTLEDLLMGTLGIVCPICSQKP       98
A4        CDSTLRLCVQSTHVDIRTLEDLLMGTLGIVCPICSQKP       98
B2        CDSTLRLCVQSTHVDIRTLEDLLMGTLGIVCPICSQKP       98
C1        CDSTLRLCVQSTHVDIRTLEDLLMGTLGIVCPICSQKP       98
          *************************** **********
```

C) Amino acid alignments of E6 HPV-18 variants

```
Sublineage          Protein sequence of E6 HPV-18                          Position

A1      MARFEDPTRRPYKLPDLCTELNTSLQDIEITCVYCKTVLELTEVFEFAFKDLFVVYRDSI 60
A2      MARFEDPTRRPYKLPDLCTELNTSLQDIEITCVYCKTVLELTEVFEFAFKDLFVVYRDSI 60
A4      MARFEDPTRRPYKLPDLCTELNTSLQDIEITCVYCKTVLELTEVFEFAFKDLFVVYRDSI 60
A5      MARFEDPTRRPYKLPDLCTELNTSLQDIEITCVYCKTVLELTEVFEFAFKDLFVVYRDSI 60
C1      MARFEDPTRRPYKLPDLCTELNTSLQDIEITCVYCKTVLELTEVFEFAFKDLFVVYRDSI 60
A3      MARFEDPTRRPYKLPDLCTELNTSLQDIEITCVYCKTVLELTGVFEFAFKDLFVVYRDSI 60
B1      MARFEDPTRRPYKLPDLCTELNTSLQDIEITCVYCKTVLELTEVFEFAFKDLFVVYRDSI 60
B2      MARFEDPTRRPYKLPDLCTELNTSLQDIEITCVYCKTVLELTEVFEFAFKDLFVVYRDSI 60
B3      MARFEDPTRRPYKLPDLCTELNTSLQDIEITCVYCKTVLELTEVFEFAFKDLFVVYRDSI 60
        ****************************************** *****************
```

```
Sublineage          Protein sequence of E6 HPV-18                          Position

A1      PHAACHKCIDFYSRIRELRHYSDSVYGDTLEKLTNTGLYNLLIRCLRCQKPLNPAEKLRH 120
A2      PHAACHKCIDFYSRIRELRHYSDSVYGDTLEKLTNTGLYNLLIRCLRCQKPLNPAEKLRH 120
A4      PHAACHKCIDFYSRIRELRHYSDSVYGDTLEKLTNTGLYNLLIRCLRCQKPLNPAEKLRH 120
A5      PHAACHKCIDFYSRIRELRHYSDSVYGDTLEKLTNTGLYNLLIRCLRCQKPLNPAEKLRH 120
C1      PHAACHKCIDFYSRIRELRHYSDSVYGDTLEKLTNTGLYNLLIRCLRCQKPLNPAEKLRH 120
A3      PHAACHKCIDFYSRIRELRHYSDSVYGDTLEKLTNTGLYNLLIRCLRCQKPLNPAEKLRH 120
B1      PHAACHKCIDFYSRIRELRYYSDSVYGDTLEKLTNTGLYNLLIRCLRCQKPLNPAEKLRH 120
B2      PHAACHKCIDFYSRIRELRHYSDSVYGDTLEKLTNTGLYNLLIRCLRCQKPLNPAEKLRH 120
B3      PHAACHKCIDFYSRIRELRHYSDSVYGDTLEKLTNTGLYNLLIRCLRCQKPLNPAEKLRH 120
        *******************:****************************************
```

```
Sublineage          Protein sequence of E6 HPV-18                          Position

A1      LNEKRRFHNIAGHYRGQCHSCCNRARQERLQRRRETQV                       158
A2      LNEKRRFHNIAGHYRGQCHSCCNRARQERLQRRRETQV                       158
A4      LNEKRRFHNIAGHYRGQCHSCCNRARQERLQRRRETQV                       158
A5      LNEKRRFHNIAGHYRGQCHSCCNRARQERLQRRRETQV                       158
C1      LNEKRRFHNIAGHYRGQCHSCCNRARQERLQRRRETQV                       158
A3      LNEKRRFHNIAGHYRGQCHSCCNRARQERLQRRRETQV                       158
B1      LNEKRRFHKIAGHYRGQCHSCCNRARQERLQRRRETQV                       158
B2      LNEKRRFHKIAGHYRGQCHSCCNRARQERLQRRRETQV                       158
B3      LNEKRRFHKIAGHYRGQCHSCCNRARQERLQRRRETQV                       158
        ********:*****************************
```

D) Amino acid alignments of E7 HPV-18 variants

```
Sublineage          Protein sequence of E6 HPV-18                          Position

A1      MHGPKATLQDIVLHLEPQNEIPVDLLCHEQLSDSEEENDEIDGVNHQHLPARRAEPQRHT 60
A2      MHGPKATLQDIVLHLEPQNEIPVDLLCHEQLSDSEEENDEIDGVNHQHLPARRAEPQRHT 60
A3      MHGPKATLQDIVLHLEPQNEIPVDLLCHEQLSDSEEENDEIDGVNHQHLPARRAEPQRHT 60
A4      MHGPKATLQDIVLHLEPQNEIPVDLLCHEQLSDSEEENDEIDGVNHQHLPARRAEPQRHT 60
A5      MHGPKATLQDIVLHLEPQNEIPVDLLCHEQLSDSEEENDEIDGVNHQHLPARRAEPQRHT 60
C1      MYGPKATLQDIVLHLEPQNEIPVDLLCHEQLSDSEEENDEIDGVNHQHLPARRAEPQRHT 60
B3      MYGPKATLQDIVLHLEPQNEIPVDLLCHEQLSDSEEENDEIDGVNHQHLPARRAEPQRHT 60
B1      MYGPKATLQDIVLHLEPQNEIPVDLLCHEQLSDSEEENDEIDGVNHQHLPARRAEPQRHT 60
B2      MYGPKATLQDIVLHLEPQNDIPVDLLCHEQLSDSEEENDEIDGVNHQHLPARRAEPQRHT 60
        *:*****************:****************************************
```

Sublineage	Protein sequence of E6 HPV-18	Position
A1	MLCMCCKCEARIELVVESSADDLRAFQQLFLNTLSFVCPWCASQQ	105
A2	MLCMCCKCEARIELVVESSADDLRAFQQLFLNTLSFVCPWCASQQ	105
A3	MLCMCCKCEARIELVVESSADDLRAFQQLFLNTLSFVCPWCASQQ	105
A4	MLCMCCKCEARIELVVESSADDLRAFQQLFLNTLSFVCPWCASQQ	105
A5	MLCMCCKCEARIELVVESSADDLRAFQQLFLNTLSFVCPWCASQQ	105
C1	MLCMCCKCEARIELVVESSADDLRAFQQLFLNTLSFVCPWCASQQ	105
B3	MLCMCCKCEARIELVVESSADDLRAFQQLFLKTLSFVCPWCASQQ	105
B1	MLCMCCKCEARIELVVESSADDLRAFQQLFLSTLSFVCPWCASQQ	105
B2	MLCMCCKCEARIELVVESSADDLRAFQQLFLSTLSFVCPWCASQQ	105
	*******************************.*************	

3. NCBI accession number of each HPV-16 and -18 variants that were used for alignments according to Papillomavirus Episteme (PaVE) portal [69].

HPV-16 SUBLINEAGES	NCBI ACCESSION NUMBER
**A1**	NC_001526
**A2**	AF536179
**A3**	HQ644236
**A4**	AF534061
**B1**	AF536180
**B2**	HQ644298
**B3**	KU053910
**B4**	KU053914
**C1**	AF472509
**C2**	HQ644244
**C3**	KU053921
**C4**	KU053922
**D1**	HQ644257
**D2**	AY686579
**D3**	AF402678
**D4**	KU053933

HPV-18 SUBLINEAGES	NCBI ACCESSION NUMBER
A1	AY262282
A2	EF202146
A3	EF202147
A4	EF202151
A5	GQ180787
B1	EF202155
B2	KC470225
B3	EF202152
C1	KC470229
